# When to be temperate: on the fitness benefits of lysis vs. lysogeny

**DOI:** 10.1093/ve/veaa042

**Published:** 2020-05-22

**Authors:** Guanlin Li, Michael H Cortez, Jonathan Dushoff, Joshua S Weitz

**Affiliations:** Interdisciplinary Graduate Program in Quantitative Biosciences, Georgia Institute of Technology, Atlanta, GA 30332, USA; School of Physics, Georgia Institute of Technology, Atlanta, GA 30332, USA; Department of Biological Science, Florida State University, Tallahassee, FL 32306, USA; Department of Biology, McMaster University, Hamilton, ON L8S 4L8, Canada; Department of Mathematics and Statistics, McMaster University, Hamilton, ON L8S 4L8, Canada; M. G. DeGroote Institute for Infectious Disease Research, McMaster University, Hamilton, ON L8S 4L8, Canada; School of Physics, Georgia Institute of Technology, Atlanta, GA 30332, USA; School of Biological Sciences, Georgia Institute of Technology, Atlanta, GA 30332, USA

**Keywords:** ecology, epidemiology, invasion fitness, viral ecology, microbial ecology, mathematical modeling

## Abstract

Bacterial viruses, that is ‘bacteriophage’ or ‘phage’, can infect and lyse their bacterial hosts, releasing new viral progeny. In addition to the lytic pathway, certain bacteriophage (i.e. ‘temperate’ bacteriophage) can also initiate lysogeny, a latent mode of infection in which the viral genome is integrated into and replicated with the bacterial chromosome. Subsequently, the integrated viral genome, that is the ‘prophage’, can induce and restart the lytic pathway. Here, we explore the relationship among infection mode, ecological context, and viral fitness, in essence asking: when should viruses be temperate? To do so, we use network loop analysis to quantify fitness in terms of network paths through the life history of an infectious pathogen that start and end with infected cells. This analysis reveals that temperate strategies, particularly those with direct benefits to cellular fitness, should be favored at low host abundances. This finding applies to a spectrum of mechanistic models of phage–bacteria dynamics spanning both explicit and implicit representations of intra-cellular infection dynamics. However, the same analysis reveals that temperate strategies, in and of themselves, do not provide an advantage when infection imposes a cost to cellular fitness. Hence, we use evolutionary invasion analysis to explore when temperate phage can invade microbial communities with circulating lytic phage. We find that lytic phage can drive down niche competition amongst microbial cells, facilitating the subsequent invasion of latent strategies that increase cellular resistance and/or immunity to infection by lytic viruses—notably this finding holds even when the prophage comes at a direct fitness cost to cellular reproduction. Altogether, our analysis identifies broad ecological conditions that favor latency and provide a principled framework for exploring the impacts of ecological context on both the short- and long-term benefits of being temperate.

## 1. Introduction

Viruses of microbes are ubiquitous in natural systems, for example densities of virus particles typically exceed 10^7^ per ml in marine systems and 10^8^ per g in soils. Viral infections can transform the fate of target cells, populations, and associated ecosystems (Bergh et al. 1989; Weinbauer 2004; Suttle 2005; Weitz 2015; Breitbart et al. 2018). Bacteriophage infections can lead to lysis and death of the infected cell, and new infections by progeny virus particles can drive down microbial populations leading to endogenous oscillations in population densities (Levin, Stewart, and Chao 1977; Lenski 1984). However, for many bacteriophage, lysis is not the only possible infection outcome.

Infection by temperate bacteriophage such as phage *λ*, *μ*, and P22 can lead to cell lysis or lysogeny (Lwoff 1953; Bertani 2004). The ‘decision’ process associated with lysis and lysogeny has been termed a genetic switch (Ptashne 2004). For example, in phage *λ*, the switch is modulated by a bidirectional promoter that controls expression of regulatory proteins whose stochastic expression and feedback culminates in either lysis or lysogeny (e.g. refer to (Golding 2018) for a recent review). Notably, the probability of initiating lysogeny and the rate of spontaneous induction are both evolvable traits (Berngruber et al. 2013). For context, induction denotes the excision of the prophage from the bacterial genome and the initiation of the lytic pathway. The evolvability of quantitative traits associated with temperate phage raises the question: how do the benefits of lysogeny vary with ecological conditions?

Prior hypotheses had suggested that temperate phage have an evolutionary advantage when few hosts are available and extracellular virion decay rates are high (Levin and Lenski 1983). In 1984, Frank Stewart and Bruce Levin addressed this question by analyzing nonlinear dynamics models of nutrients, cells, virulent phage, and temperate phage (Stewart and Levin 1984). However, Stewart and Levin reported that ‘in spite of the intuitive appeal of this low density hypothesis, we are unable to obtain solutions consistent with it using the model presented here’. Instead, they introduced external oscillations in resource supply rates to identify regimes in which both virulent and temperate bacteriophage could coexist. Moreover, by analyzing population abundances as a proxy for evolutionary success, the ‘advantage’ of a temperate vs. obligately lytic strategy was compared in terms of relative abundances of virus particles and infected cells. Such weightings are seemingly arbitrary and do not stem from an evolutionary framework.

Here, we re-assess the benefits of being temperate by asking the question: under what ecological conditions can temperate phage potentially invade microbial communities, including those without and with circulating lytic phage? First, we adapt a cell-centric metric of viral invasion fitness to the ecological dynamics of viruses and their microbial hosts (Berngruber et al. 2013; Gandon 2016; Weitz et al. 2019). Related work has shown that threshold criterion—typically used in the study of epidemiological dynamics—can be used to identify conditions underlying the feasible invasion of a microbial population by lytic and/or temperate phage (Berngruber et al. 2013; Gandon 2016; Wahl et al. 2018; Weitz et al. 2019). However, the resulting conditions are often complicated algebraic expressions that defy biological interpretation. Here, using a novel application of Levins’ network loop analysis (Levins 1974), we show that complicated algebraic expressions for viral fitness can be biologically interpreted in terms of purely horizontal, vertical, and mixed transmission pathways. In doing so, we show that temperate strategies can invade virus-free environments when susceptible densities are relatively low and when the integrated prophage confers direct fitness benefits to cellular growth and survival (consistent with Berngruber et al. 2013; Wahl et al. 2018, albeit here we place a greater emphasis on the relationship between ecological context and invasion). However, prophage can sometimes impose a cost to cellular growth and survival. Hence, we use evolutionary invasion analysis to identify when temperate phage can successfully invade a community including bacteria and circulating lytic phage. As we show, lytic phage can drive down microbial cell densities so as to enable invasion by temperate phage that confer protection against subsequent infection. This result holds even when the temperate phage provide no direct benefit or even impose a cost to cellular growth. Overall, this analysis provides a theoretical framework for identifying near-term ‘solutions’ (sensu Stewart and Levin 1984) to the problem of when to be temperate in an ecological context and an exploration of conditions for coexistence between lytic and temperate phage (complementary to studies of the long-term evolution of temperate strategies Wahl et al. 2018).

## 2 Results

### 2.1 Nonlinear, population model of temperate phage dynamics

**Figure 1.**
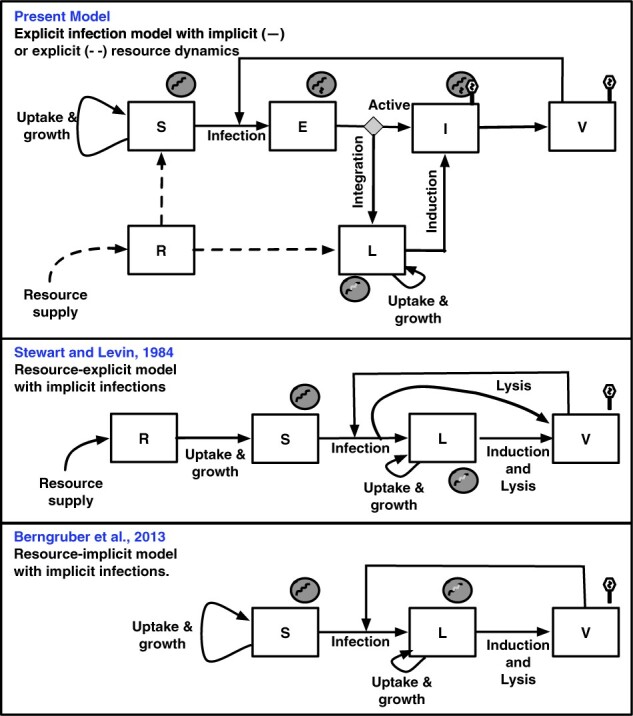
Schematic of nonlinear dynamics population models of temperate phage. (Top) The explicit infection model with implicit (–) or explicit (- -) resource dynamics (developed here). (Middle) Resource-explicit model with implicit infections (Stewart and Levin 1984). (Bottom) Resource-implicit model with implicit infections (Berngruber et al. 2013). The governing equations for each model are presented in the Main Text (Top), Section 4 (Middle and Bottom), with extended analysis in Supplementary Appendix A.

We begin by considering the dynamics of temperate phage in a nonlinear population model that includes explicit representation of infections including cells that are either susceptible (*S*), exposed (*E*), actively infected (*I*), or lysogens (*L*), as well as virus particles (*V*), see the top panel of Fig. 1. The exposed cells are cells that have been infected but the virus has not yet committed to either the lytic pathway (turning it into an actively infected, *I*, cell) or the lysogenic pathway (turning it into a lysogen, *L*). In this model, the life history traits of a temperate phage are defined by two evolvable parameters: *p*, the probability a virus enters the lysogenic pathway and *γ*, the induction rate after a virus enters the lysogenic pathway. We represent this model in terms of a system of nonlinear ordinary differential equations (ODEs):

(1) $$\begin{array}{ll} \dot{S} & =\overset{\text{growth}}{\overset{︷}{b_{S}S}}-\overset{\text{infection}}{\overset{︷}{\phi SV}}-\overset{\text{decay}}{\overset{︷}{d_{S}S}}\\ \dot{E} & =\overset{\text{infection}}{\overset{︷}{\phi SV}}-\overset{\text{transition}}{\overset{︷}{\lambda E}}-\overset{\text{decay}}{\overset{︷}{d_{E}E}}\\ \dot{L} & =\overset{\text{lysogenic infection}}{\overset{︷}{p\lambda E}}+\overset{\text{growth}}{\overset{︷}{b_{L}L}}-\overset{\text{induction}}{\overset{︷}{\gamma L}}-\overset{\text{decay}}{\overset{︷}{d_{L}L}}\\ \dot{I} & =\overset{\text{lytic infection}}{\overset{︷}{(1-p)\lambda E}}+\overset{\text{induction}}{\overset{︷}{\gamma L}}-\overset{\text{lysis}}{\overset{︷}{\eta I}}-\overset{\text{decay}}{\overset{︷}{d_{I}I}}\\ \dot{V} & =\overset{\text{burst}}{\overset{︷}{\beta \eta I}}-\overset{\text{infection}}{\overset{︷}{\phi NV}}-\overset{\text{decay}}{\overset{︷}{mV}}. \end{array}$$

In this model, ϕ is the adsorption rate, $d_{S},d_{E},d_{L}$ and $d_{I}$ are the cellular death rates of susceptible cells, exposed infected cells, lysogens, and lytic-fated infected cells, respectively, λ is the transition rate from exposed cells to the fate determined cells, *p* is the probability of lysogeny, *γ* is the induction rate, *η* is the lysis rate, *β* is the burst size, and *m* is the virion decay rate. The growth rates of susceptible hosts and lysogens are denoted by *b_S_* and *b_L_*, respectively. For the resource-implicit model, the growth rates are

(2) $$\begin{array}{c} b_{S}(N)=r_{S}\left(1-\frac{N}{K}\right),\;b_{L}(N)=r_{L}\left(1-\frac{N}{K}\right) \end{array},$$

where N=S+E+L+I is the population density of total cells. Parameters $r_{S}$ and $r_{L}$ denote the maximal cellular growth rates of susceptible cells and lysogens, *K* is the carrying capacity. For the resource-explicit model, the growth rates of susceptible hosts and lysogens are

(3) $$\begin{array}{c} b_{S}(R)=(1-\alpha_{s})\psi (R),\;b_{L}(R)=\psi (R) \end{array},$$

where *R* is the resource density and $\psi (R)=\mu_{\text{max}}R/(R_{\text{in}}+R)$ is the Monod equation. For the resource-explicit model, we add one additional equation to describe the dynamics of the resources,

(4) $$\begin{array}{l} \dot{R}=\overset{\text{influx}}{\overset{︷}{J}}-\overset{\text{decay}}{\overset{︷}{d_{R}R}}-\overset{\text{uptake of nutrients}}{\overset{︷}{f(R,S,L)}} \end{array},$$

where $f(R,S,L)=e\psi (R)\left(L+(1-\alpha_{s})S\right)$ denotes the cumulative uptake of nutrients by all cells. Parameters $\mu_{\text{max}}$ and $R_{\text{in}}$ are the maximal cellular growth rate and the half-saturation constant, *J* and $d_{R}$ are the influx and decay rates of resources, *e* is the conversion efficiency, *α_s_* is the selection coefficient that measures the relative difference in the reproductive output between lysogens and susceptible cells. Model 1 allows us to analyze the dynamics of viruses with different life history strategies. Here, a viral strategy is defined by different combinations of the trait values (*p*, *γ*). In the two-dimensional viral strategy space we denote the purely lytic strategy as *p *=* *0 (for which the value of *γ* is irrelevant, and assumed to be *γ*_max_ for convenience), and the purely lysogenic strategy as $p=1,\gamma =\gamma_{\text{min}}$, where $\gamma_{\text{min}}>0$. Here, a temperate viral strategy is characterized by 0<p≤1 and $\gamma \geq \gamma_{\text{min}}$. This model extends earlier proposals to model temperate phage via implicit infections (i.e. without an explicit state that demarcates the decision between lysis and lysogeny), either with explicit resource dynamics (Stewart and Levin 1984) or with implicit resource dynamics (Berngruber et al. 2013). Critically, all of these models include the possibility of both vertical and horizontal transmission of phage genomes. These models also set the basis for evaluating temperate phage invasion given variation in disease-free environment conditions, that is given the equilibrium concentrations of $S^{*}$ in resource-implicit cases or $\left(R^{*},S^{*}\right)$ in resource-explicit cases.

### 2.2 Viral invasion analysis

Here, we show how our model can be used to compute viral invasion fitness for viruses with different lysogeny–lysis strategies, that is combinations of trait values (*p*, *γ*). The spread of temperate viruses in a parasite-free environment can be analyzed in terms of the basic reproduction number $R_{0}$, which denotes the average number of new infected cells produced by a single (typical) infected cell and its progeny virions in an otherwise susceptible population (Berngruber et al. 2013; Gandon 2016; Weitz et al. 2019). When $R_{0}$ is greater than 1, the pathogen will spread and when $R_{0}$ is less than 1, the pathogen will not spread. The next-generation matrix (NGM) approach can be used to calculate $R_{0}$ (Diekmann, Heesterbeek, and Roberts 2010). The NGM represents the expected progeny for transitions between all combinations of ‘epidemiological birth states’, that is states that can produce newly infected hosts cells. In our model, states *E* and *L* are the only epidemiological birth states and epidemiological births arise due to infection of cells by virions and by the division of lysogenic cells. The largest positive eigenvalue of the NGM is equivalent to $R_{0}$. The NGM for model 1 is the 2 × 2 matrix Φ:

(5) $$\begin{array}{l} \Phi =\left[\begin{array}{cc} \overset{E\to I\to V\to E}{\overset{︷}{(1-p)R^{\text{hor}}}}+\overset{E\to L\to I\to V\to E}{\overset{︷}{p\tilde{\gamma }R^{\text{hor}}}} & \overset{L\to I\to V\to E}{\overset{︷}{R^{\text{hor}}\left(\tilde{\gamma }/\tilde{\lambda }\right)}}\\ \overset{E\to L\to L}{\overset{︷}{p\tilde{\lambda }R^{\text{ver}}(1-\tilde{\gamma })}} & \overset{L\to L}{\overset{︷}{R^{\text{ver}}(1-\tilde{\gamma })}} \end{array}\right] \end{array}.$$

Here, $\tilde{\gamma }=\gamma /(\gamma +d_{L})$ is the probability induction occurs before cell death, $\tilde{\lambda }=\lambda /(\lambda +d_{E})$ is the probability that exposed cells enter the lysogenic pathway before cell death, $R^{\text{hor}}$ and $R^{\text{ver}}$ are the basic reproduction numbers of purely lytic phage (*p *=* *0) and purely lysogenic phage (p=1,γ=0), respectively:

(6) $$R^{\text{hor}}=\overset{E\to I}{\overset{︷}{\frac{\lambda }{\lambda +d_{E}}}}\overset{I\to V}{\overset{︷}{\left(\frac{\beta \;\eta }{\eta +d_{I}}\right)}}\overset{V\to E}{\overset{︷}{\left(\frac{\phi S^{*}}{\phi S^{*}+m}\right),}}$$

(7) $$R^{\text{ver}}=\overset{L\to L}{\overset{︷}{\frac{b_{L}}{d_{L}}}},$$

where $S^{*}=K(1-d_{S}/r_{S})$ is the susceptible host density in the virus-free environment. The vertical contribution to fitness is modulated by infected cell growth rates, $b_{L}$, which in the resource-implicit and resource-explicit systems are $r_{L}(1-S^{*}/K)$ and $\psi (R^{*})$, respectively, where $R^{*}$ is the resource concentration in the virus-free environment.

Each entry $\Phi_{ij}$ in the NGM represents the expected number of new infected individuals in epidemiological birth state *i* (either *L* or *E*), generated by one infected individual at epidemiological birth state *j* (either *L* or *E*), accounting for new infections that arise via the lytic and lysogenic pathways. For example, $\Phi_{11}$ accounts for the expected number of exposed cells *E* produced by a single exposed cell. The single exposed cell *E* has a probability $(1-p)\tilde{\lambda }$ of entering the *I* state, of which a fraction $\eta /(\eta +d_{I})$ of infected cells will release viruses. Each infected cell produces *β* free virus particles given successful lysis. The factor $\phi S^{*}/(\phi S^{*}+m)$ denotes the probability that a free virus particle is adsorbed into a susceptible cell before it decays. As such, $(1-p)R^{\text{hor}}$ is the expected number of newly infected exposed cells produced by a single exposed cell via a purely lytic pathway. Similarly, an exposed cell *E* has a probability $p\tilde{\lambda }$ of entering the *L* state and being induced from the lysogenic cell *L* to infected cell *I*. Thus, a single exposed cell produces $p\tilde{\gamma }R^{\text{hor}}$ newly infected exposed cells on average after a sequence of integration, induction, and lysis events. Altogether, the expected number of exposed cells produced by a single exposed cell is $\Phi_{11}=(1-p)R^{\text{hor}}+p\tilde{\gamma }R^{\text{hor}}$. The other entries in the NGM can be interpreted similarly; the transmission pathways are labeled in Equation (5) for each entry of the NGM.

In the event that phage cannot induce, that is *γ *= 0, then the basic reproduction number reduces to:

(8) $$R_{0}=\text{max}(R^{\text{ver}},(1-p)R^{\text{hor}}),$$

which corresponds to the viral invasion fitness associated with either lysis or lysogeny, but not both, equivalent to the finding in Weitz et al. (2019).

The use of a cell-centric metric enables direct comparisons of lytic and lysogenic pathways, that is even in the absence of virion production from lysogens. In the general case where induction is possible, that is γ>0, then, the basic reproduction number $R_{0}$ becomes:

(9) $$\begin{array}{c} R_{0}=\frac{1}{2}\left(\text{Tr}(\Phi )+\sqrt{\text{Det}{\left(\Phi \right)}^{2}-4\;\text{Det}(\Phi )}\right), \end{array}$$

where the trace Tr(Φ) is:

(10) $$\text{Tr}(\Phi )=R^{\text{hor}}\tilde{\gamma }p+(1-p)R^{\text{hor}}+(1-\tilde{\gamma })R^{\text{ver}},$$

and the determinant Det(Φ) is:

(11) $$\text{Det}(\Phi )=R^{\text{hor}}R^{\text{ver}}(1-p)(1-\tilde{\gamma }).$$

Notably, this formula for the basic reproduction number applies to all model variants of temperate phage dynamics listed in Fig. 1, see Supplementary Appendix B2. However, Equation (9) poses multiple challenges for interpreting $R_{0}$ not only for phage, but also for generalized cases of host–pathogen dynamics with multiple transmission modes (van den Driessche 2017). It is the interpretation problem that we address next.

### 2.3 Interpreting $R_{0}$ using Levins’ loop analysis

Here, we use Levins’ loop analysis (Levins 1974) to interpret the basic reproduction number $R_{0}$ arising in each of the models depicted in Fig. 1. Levins’ loop analysis was developed for the analysis of feedback in ecological networks (Levins 1974)—which we adapt to the study of the network of paths in the life history of an infectious pathogen. Loop analysis has been used previously to interpret $R_{0}$ for discrete-time stage structured population dynamics (de Camino-Beck and Lewis 2007). In another instance, previous work (Rueffler and Metz 2013) focused on applications of loop analysis to models where the NGM only has a single non-zero eigenvalue whereas the NGM given by Equation (5) has multiple non-zero eigenvalues. In the present context, we define a one-generation loop as the collection of paths that start in one infection class and ends in the same class without revisiting any classes. Next, we define a joint two-generation loop as a pair of one-generation loops that start and end in the same class. Finally, we define a disjoint two-generation loop as a pair of one-generation loops whose pathways do not share an epidemiological birth state.

**Figure 2.**
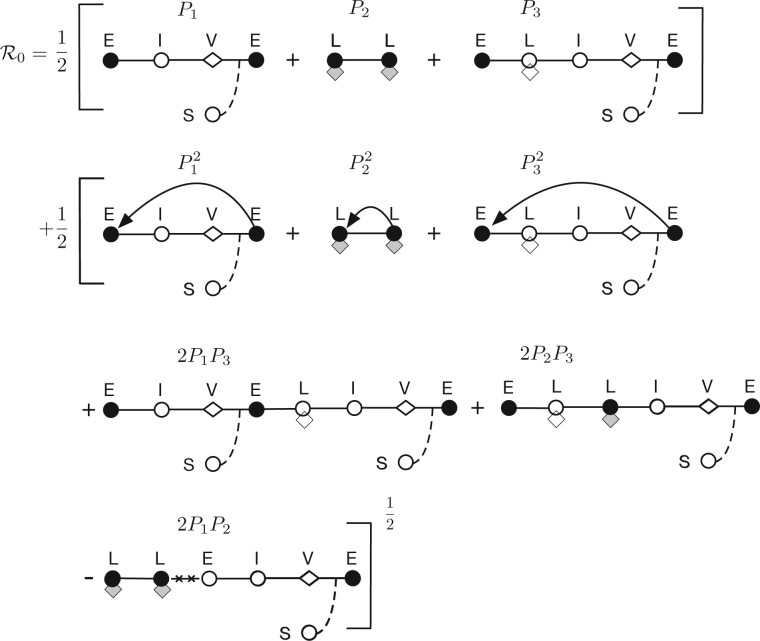
Loop-based interpretation of the basic reproduction number for horizontal and vertical transmission of temperate phage. Closed circles denote epidemiological births, open circles represent infected cell transitions, diamonds represent virus particles, and lysogens are denoted using a hybrid symbol (denoting the presence of an integrated viral genome). Solid lines denote transitions between states, lines with arrows denote a repeat of the same loop, dashed lines denote interactions with uninfected cells, and crossed out lines (-x-x-) denote non-feasible transitions.

Using these definitions, we denote the lytic loop as ①. In this loop, a newly infected individual in state *E* passes to state *I* and releases virions that lead to new hosts entering the *E* state. The first loop in Fig. 2 shows the pathway from *E* to *I* to *V* and then back to *E*. We denote the lysogenic loop as ②. In this loop, lysogens are reproduced during the lifespan of an individual lysogen. The second loop in Fig. 2 shows the pathway from *L* to *L*. We denote the lyso–lytic loop as ③. In this loop, a newly infected individual in state *E* passes to state *L* and then induces from *L* to *I* (without dividing), then releases virions that lead to new hosts entering the *E* state. The third loop in Fig. 2 shows the pathway from *E* to *L* to *I* to *V* and then back to *E*.

We can compute a reproduction number for each loop, that is the expected number of new infections that arises from each loop. We denote the reproduction number of the lytic loop ① as $P_{1}$, the reproduction number of the lysogenic loop ② as $P_{2}$ and the reproduction number of the lyso–lytic loop ③ as $P_{3}$. The reproduction numbers of one-generation loops can be directly read-off from the NGM Φ, Equation (5),

(12) $$\begin{array}{ll} & ①\;\text{Lytic loop}:\overset{E\to I\to V\to E}{\overset{︷}{(1-p)R^{\text{hor}}}}=P_{1}\\ & ②\;\text{Lysogenic loop}:\overset{L\to L}{\overset{︷}{(1-\tilde{\gamma })R^{\text{ver}}}}=P_{2}\\ & ③\;\text{Lyso}-\text{lytic loop}:\overset{E\to L\to I\to V\to E}{\overset{︷}{p\tilde{\gamma }R^{\text{hor}}}}=P_{3}. \end{array}$$

Among all the pairs of one-generation loops, there are seven joint two-generation loops with permutations, for example ①⊕①, ②⊕②, ③⊕③, ①⊕③, ③⊕①, ②⊕③ and ③⊕②, and two disjoint pairs of loops, for example ①⊕② and ②⊕①. Using the above, Equation (9) for $R_{0}$ can be rewritten in the following form:

(13) $$\begin{array}{cc} R_{0} & =\frac{1}{2}\left(P_{1}+P_{2}+P_{3}\right)\\ & +\frac{1}{2}{\left(P_{1}^{2}+P_{2}^{2}+P_{3}^{2}+2P_{1}P_{3}+2P_{2}P_{3}-2P_{1}P_{2}\right)}^{\frac{1}{2}}. \end{array}$$

Notice that $P_{1}+P_{2}+P_{3}$ represents the sum of the one-generation loops and the terms in the square root represent the sum of the joint two-generation loops discounted by the disjoint loops. The contributions from all two-generation loops are discounted by the 1/2 exponent because the basic reproductive number focuses on reproductive output after one generation. Figure 2 shows the equivalency between Equation (13) and the loop-based interpretation.

Notably, the calculations of $R_{0}$ for all temperate phage models in Fig. 1 can be expressed in the form of Equation (13). Table 1 summarizes the equivalencies of the component calculations for $R_{0}$ in each of the models given a lytic loop, lysogenic loop, and a mixed loop. Moreover, this loop interpretation also applies to reduced versions of these models as long as they retain two epidemiological birth states (e.g. the *SILV*-system, as analyzed in Weitz et al. (2019)). Although the models differ in their mechanistic details, each shares two evolvable traits: P, the probability of entering lysogenic state after infection and *γ*, the rate of induction from a lysogenic state (Refardt and Rainey 2009). This similarity in form suggests that the dependency for invasion on the temperate traits *p* and *γ* may also transcend model details.

**Table 1.** Loop-based $R_{0}$ calculations for temperate phage models.

Temperate phage dynamics	Lytic loop *P*_1_	Lysogenic loop *P*_2_	Lyso–lytic loop *P*_3_
Resource-explicit model with implicit infections (Stewart and Levin 1984)	$\overset{V\to V}{\overset{︷}{(1-p)\left(\frac{\beta \phi S^{*}}{\rho }\right)}}$	$\overset{L\to L}{\overset{︷}{\frac{\psi (R^{*})}{\gamma +\rho +\nu }}}$	$\overset{L\to V}{\overset{︷}{\left(\frac{\gamma }{\gamma +\rho +\nu }\right)}}\overset{V\to L}{\overset{︷}{\left(\frac{\beta \phi S^{*}}{\rho }\right)p}}$
Resource-implicit model with implicit infections (Berngruber et al. 2013)	$\overset{V\to V}{\overset{︷}{(1-p)\left(\frac{b\beta \phi S^{*}}{\phi S^{*}+m}\right)}}$	$\overset{L\to L}{\overset{︷}{\frac{r_{L}\delta \left(1-S^{*}/K\right)}{m+\gamma }}}$	$\overset{L\to V}{\overset{︷}{\left(\frac{\gamma }{\gamma +m}\right)}}\overset{V\to L}{\overset{︷}{\left(\frac{b\beta \phi S^{*}}{\phi S^{*}+m}\right)p}}$
Resource-explicit model with explicit infections (Present)	$\overset{E\to I}{\overset{︷}{(1-p)\left(\frac{\lambda }{\lambda +d_{E}}\right)}}\overset{I\to V}{\overset{︷}{\left(\frac{\beta \;\eta }{\eta +d_{I}}\right)}}\overset{V\to E}{\overset{︷}{\left(\frac{\phi S^{*}}{\phi S^{*}+m}\right)}}$	$\overset{L\to L}{\overset{︷}{\frac{r_{L}(1-S^{*}/K)}{\gamma +d_{L}}}}$	$\overset{E\to L}{\overset{︷}{p\left(\frac{\lambda }{\lambda +d_{E}}\right)}}\overset{L\to I}{\overset{︷}{\left(\frac{\gamma }{\gamma +d_{L}}\right)}}\overset{I\to V}{\overset{︷}{\left(\frac{\beta \;\eta }{\eta +d_{I}}\right)}}\overset{V\to E}{\overset{︷}{\left(\frac{\phi S^{*}}{\phi S^{*}+m}\right)}}$
Resource-implicit model with explicit infections (Present)	$\overset{E\to I}{\overset{︷}{(1-p)\left(\frac{\lambda }{\lambda +d_{E}}\right)}}\overset{I\to V}{\overset{︷}{\left(\frac{\beta \;\eta }{\eta +d_{I}}\right)}}\overset{V\to E}{\overset{︷}{\left(\frac{\phi S^{*}}{\phi S^{*}+m}\right)}}$	$\overset{L\to L}{\overset{︷}{\frac{\psi (R^{*})}{\gamma +d_{L}}}}$	$\overset{E\to L}{\overset{︷}{p\left(\frac{\lambda }{\lambda +d_{E}}\right)}}\overset{L\to I}{\overset{︷}{\left(\frac{\gamma }{\gamma +d_{L}}\right)}}\overset{I\to V}{\overset{︷}{\left(\frac{\beta \;\eta }{\eta +d_{I}}\right)}}\overset{V\to E}{\overset{︷}{\left(\frac{\phi S^{*}}{\phi S^{*}+m}\right)}}$

### 2.4 Feasible invasion strategies

We systematically analyze the dependency of the viral invasion fitness $R_{0}$ on the viral ‘strategy’, that is the combination of temperate traits (p,γ), for all four temperate phage models depicted in Fig. 1. A strategy (p,γ) is feasible if $R_{0}(p,\gamma )>1$. We conduct this invasion analysis to distinguish when a temperate strategy is feasibly and/or obligately invasible. For feasible invasibility, we identify the ecological conditions in which a temperate viral strategy can invade in a completely susceptible host population (i.e. which combination of traits corresponds to $R_{0}(p,\gamma )>1$). For obligate invasibility, we identify the ecological conditions in which a temperate viral strategy is required for invasion of a completely susceptible host population such that $R_{0}(p,\gamma )>1$ for all 0<p≤1 whereas the purely lytic strategy cannot invade, that is $R_{0}(0,\gamma )<1$.

**Figure 3.**
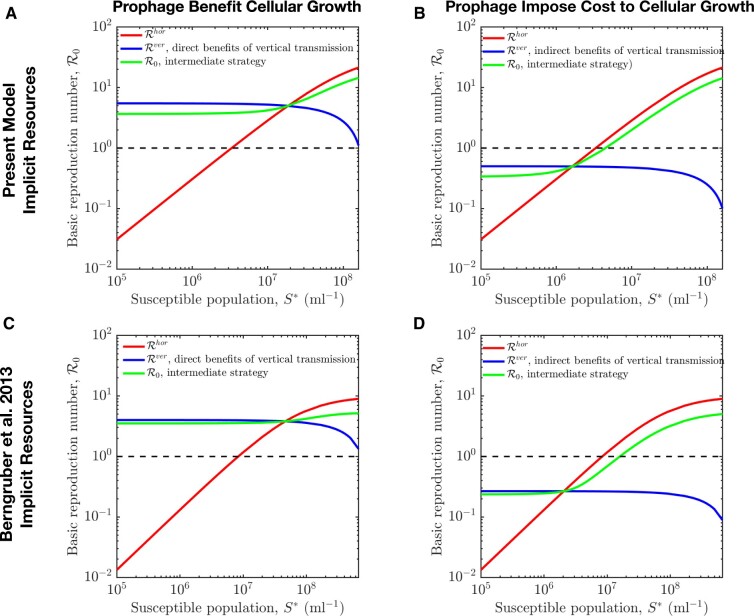
Feasible invasion for viral strategies given variation in susceptible host densities. In panels A and C, prophage provide direct benefit to cellular fitness, that is $R^{\text{ver}}>1$ given variation in susceptible host densities. In contrast, panels B and D show the case that prophage impose cost to cellular fitness, that is $R^{\text{ver}}<1$ given variation in susceptible host densities. For the intermediate strategy, the probability of lysogeny is *p* = 0.5 and the induction rate is γ=0.1/h, see model details and relevant parameters in Sections 2 and 4, Supplementary Appendixes A and E.

**Figure 4.**
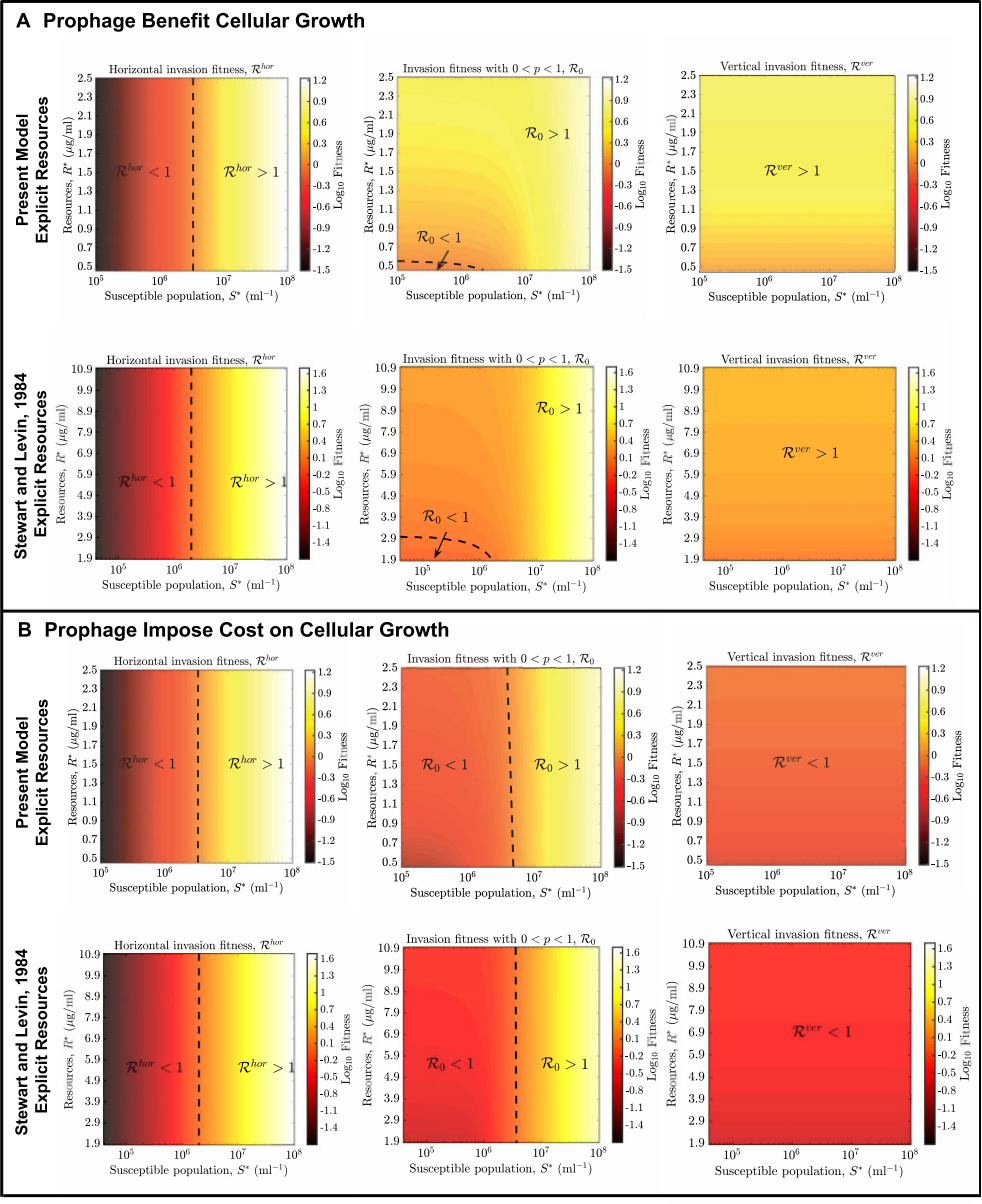
Feasible invasion for viral strategies given variation in resources and susceptible host densities. (A) Prophage provide direct benefit to cellular fitness, that is $R^{\text{ver}}>1$. (B) Prophage impose cost to cellular fitness, that is $R^{\text{ver}}<1$. The probability of lysogeny is *p *=* *0.5 and the induction rate is γ=0.1/h for the intermediate strategy. Additional model details and relevant parameters are in Section 4, Supplementary Appendixes A and E.

To begin, it is useful to delineate the bounds to $R_{0}$ in special cases. As shown in Supplementary Appendix C2, we find that in the case of a resource-implicit model the purely lytic strategy maximizes $R_{0}$ when $R^{\text{hor}}>R^{\text{ver}}$, that is more newly infected cells are produced through the lytic pathway than the lysogenic pathway. In contrast, the purely lysogenic strategy maximizes $R_{0}$ when $R^{\text{hor}}<R^{\text{ver}}$, that is more newly infected cells are produced through the lysogenic pathway than the lytic pathway. Notably, $R^{\text{ver}}$ decreases with susceptible host density $S^{*}$ while $R^{\text{hor}}$ increases with susceptible host density $S^{*}$. Hence, $R_{0}$ is higher for temperate phage until $S^{*}$ is sufficiently high that $R_{0}$ is higher for lytic phage. For the resource-explicit models, the virus-free environment is represented by the susceptible host density ($S^{*}$) and resource density ($R^{*}$). In the $S^{*}$–$R^{*}$ plane, there is a critical transition curve (*S*_c_, *R*_c_) defined by $R^{\text{hor}}(S_{c})=R^{\text{ver}}(R_{c})$ where the strategy associated with maximal fitness (i.e. that maximizes $R_{0}$) switches from purely lysogenic to purely lytic (see Supplementary Appendix C2). This analysis reveals that a purely lysogenic strategy is favored given low cell abundances and high resources (above the $\left(S_{c},R_{c}\right)$ curve) and a purely lytic strategy is favored given high cell abundances and low resources (below the $\left(S_{c},R_{c}\right)$ curve). We remark that maximizing $R_{0}$ is not equivalent to long-term evolutionary success, for example when mutant viruses must contend with environments set by a resident. Nonetheless, calculating bounds on $R_{0}$ helps to identify the basis for feasible strategies such that viruses can become a potential resident. Analogous to the resource-implicit case, these bounds provide the basis for identifying feasible strategies, depending on whether prophage provide a direct benefit or impose a cost to cellular fitness. First, consider the case where prophage provide a direct benefit to cellular fitness, that is such that $\left(b_{L}/d_{L})>(b_{S}/d_{S}\right)$ at the virus-free equilibrium. It would seem apparent that lysogeny (and therefore temperate strategies) should enable viral invasion of an entirely susceptible host population. Indeed, a purely vertical strategy is feasible irrespective of cell density because newly produced lysogens out-compete resident cells; this is true for both resource-implicit (see Fig. 3A and C) and resource-explicit models (see Fig. 4A). In contrast, the $R^{\text{hor}}$ of a horizontal strategy increases with increasing susceptible density $S^{*}$, such that it becomes feasible at a critical value $S^{*}=S_{c}$ for resource-implicit models (see Fig. 3) or beyond a curve (*R*_c_, *S*_c_) for resource-explicit models (see Fig. 4). As a consequence, intermediate temperate strategies with 0<p<1 can also be feasible at both low and high extremes of susceptible host density and resource concentration. In this case, temperate viruses derive most of their fitness via vertical transmission when $S^{*}$ is low and via horizontal transmission when $S^{*}$ is high. In practice, we find that temperate strategies can be feasible across the entire range of host densities (and resource levels), including in circumstances where lytic strategies have $R_{0}<1$ (see Figs 3 and 4).

In contrast, using the same analysis framework we find that a purely lysogenic strategy with *p *=* *1 is not feasible when prophage impose a cost to cellular fitness, that is $\left(b_{L}/d_{L})<(b_{S}/d_{S}\right)$, at the virus-free equilibrium. This holds for both the resource-implicit and -explicit models (see Figs 3B, D, and 4B). As a result, a temperate strategy with *p *>* *0 is feasible insofar as there are sufficiently high host densities for viruses to spread predominantly via a horizontal route. In that case, a sufficient condition for temperate strategy invasion is that $(1-p)R^{\text{hor}}>1$, such that the range of ecological conditions for feasible temperate strategies is more restricted than for purely lytic strategies (see Figs 3 and 4).

In summary, temperate phage that confer a benefit to cellular fitness may invade across a wide range of ecological contexts. Yet the more interesting question raised by this analysis is: can temperate viruses that impose a direct fitness cost to cells nonetheless invade in a greater range of ecological contexts albeit when viruses are present?

### 2.5 Endemically infected states and viral invasion

We examine the extent to which temperate phage can invade environments with pre-existing (i.e. circulating) lytic viruses. We are interested specifically in the case where prophage impose a direct cost to cellular fitness, as measured in terms of the ratio of cellular reproduction to mortality in an otherwise virus-free environment. To do so, we first apply an invasion analysis (Hurford, Cownden, and Day 2010) to the resource-explicit model with explicit infections. We assume the resident strain is a purely lytic strategy $\left(p_{r}=0,\gamma_{r}=\gamma_{\text{max}}\right)$. We then determine how a strategy $\left(p_{m},\gamma_{m}\right)$ of a mutant type competes in the environment determined by the resident. We note that the resident endemic equilibrium is stable given the particular parameter set in Supplementary Appendix E.

**Figure 5.**
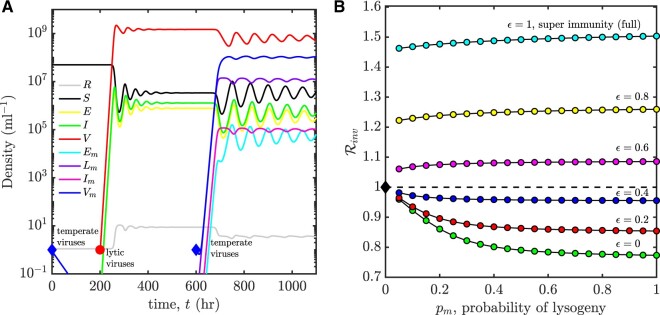
Invasion of temperate phage with varying degrees of super-infection immunity from ϵ=0 (none) to ϵ=1 (complete). (A) Example time series of population densities in the case that lytic phage can drive down microbial cell densities so as to enable invasion by temperate phage. Concretely, at time *t *=* *0 h, temperate phage with purely lysogenic strategy cannot invade virus-free environment (first blue diamond); at time *t *=* *200 h, purely lytic phage invade virus-free environment (red circle) and spread; at time *t *=* *600 h, the endemic environment set by the resident lytic strategy can be invaded by temperate phage with purely lysogenic strategy with full immunity *ϵ* = 1 (see second blue diamond). (B) The invasion fitness ($R_{\text{inv}}$) of a temperate phage strategy *p_m_* given variation in *ϵ* from *ϵ* = 0 to *ϵ* = 1, the induction rate is fixed, $\gamma_{m}=10^{-2}$/h. The model details are presented in Equation (1) and Supplementary Appendix D3, the relevant parameters are selection coefficient $\alpha_{s}=-0.42$ and decay rate of lysogens $d_{L}=0.5$/h (in the case when prophage impose cost to cellular fitness), influx rate of resources J=5.33 (μg/ml) h^−1^, see additional parameters in Supplementary Appendix E.

Consider the case when the purely lytic strategy has invaded a region with initially high susceptible host densities (high enough that a purely latent strategy would not feasibly invade—see Fig. 5A, first blue diamond). Paradoxically in this case, the environment set by the resident lytic strategy can (but not always) be invaded by a temperate strategy (i.e. $0<p_{m}\leq 1$) even if the temperate phage imposes a direct cellular fitness cost. The reason is as follows. Initially in the virus-free environment, the susceptible host density is high and resources are low. Lysis depletes susceptible hosts, which reduces niche competition between cells, thereby increasing the potential benefits of vertical transmission. This feedback implies that the vertical fitness can be higher than the horizontal fitness in the environment that lytic phage established, enabling more temperate strategies to invade (see Supplementary Appendix D for mathematical details). We also note that invasion by a mutant strain does not necessarily imply replacement of the resident strain. If the lytic virus were to become rare, then the lysogens would be out-competed by uninfected hosts, transforming the environment into one susceptible to proliferation by lytic viruses. As such, temperate viruses can invade and then coexist with lytic viruses (see Fig. 5A). These findings reveal how decomposing fitness into horizontal and vertical transmission pathways can provide mechanistic insights into eco-evolutionary outcomes (Wahl et al. 2018).

Beyond the density-dependent effect, temperate strategies that impose a fitness cost may come with another form of benefit: conferring ‘super-infection immunity’ to infection by lytic viruses. Super-infection denotes the possibility that a cell is infected by more than one virus (Abedon 2015). Super-infection immunity becomes relevant from an ecological perspective when the resident virus reaches relatively high densities, that is in the endemic context. Hence, in an effort to generalize the dynamical example in Fig. 5A, we consider a spectrum of cases in which lytic viruses are absorbed into all cells but only infect a fraction (1−ϵ) of lysogens, where *ϵ* is interpreted as the degree of super-infection immunity (see Supplementary Appendix D3). Hence, a temperate viral strategy is represented by the combination: (p,γ,ϵ). We then measure the invasion fitness $R_{\text{inv}}$ of a temperate virus strategy given variation in *ϵ* from *ϵ* = 0 (all lysogens infected by lytic viruses, no super-infection immunity) to *ϵ* = 1 (no lysogens infected by lytic viruses, full super-infection immunity). We find that temperate phage can invade an environment with hosts and a resident lytic virus insofar as they provide a critical level of super-infection immunity (see Fig. 5B).

## 3. Discussion

We have demonstrated the benefits of being temperate by exploring the dependency of viral invasion fitness on infection mode and ecological context. By integrating a cell-centric metric of viral invasion fitness (Weitz et al. 2019) and Levins’ loop analysis (Levins 1974), we derived an interpretable representation of invasion fitness ($R_{0}$) common to a series of mechanistic representations of temperate phage (see boxed Equation (13) and Fig. 2). In contrast to the previous application (Rueffler and Metz 2013) of loop analysis, our work shows that the loop interpretation of $R_{0}$ is useful even though the NGM has multiple non-zero eigenvalues. Using this form, we have shown that temperate strategies are feasible (i.e. $R_{0}>1$) for a wide range of ecological conditions, insofar as prophage provide a direct benefit to cellular fitness. In contrast, we find that temperate phage that impose a direct cost to cellular fitness can still invade environments with pre-circulating lytic viruses (particularly when they provide a critical level of super-infection immunity).

The Levins’ loop approach also enables the interpretation of temperate phage fitness, at least in the short term. In the short term, the invasion fitness $R_{0}$ depends on the infection mode as well as an ecological context, for example susceptible cell density and resources. The invasion fitness reflects contributions from vertical, horizontal, and mixed transmission pathways. The purely vertical fitness increases as resources increase or host abundances decrease, see Fig. 3. In contrast, the purely horizontal fitness increases as host abundances increase, see Fig. 3. As a consequence, we find that temperate phage feasibility is enhanced given low host abundances and high resources, whereas the purely lytic strategy is infeasible for low host abundances. This relationship is consistent with observations that lysogeny is prevalent at times of low host availability, for example in aquatic systems (Paul 2008; Payet and Suttle 2013; Warwick-Dugdale et al. 2019). In practice, nutrient concentrations co-vary with host cell abundances, and so disentangling the effects of fluxes vs. pools will be critical to translating findings here to analysis of latency in the environment (Smith, Jones II, and Smith 2005; Weitz and Dushoff 2008; Knowles et al. 2016; Knowles and Rohwer 2017; Weitz et al. 2017).

As a step toward understanding long-term evolution of temperate strategies, we considered the invasibility of endemic states, that is by examining whether temperate phage can invade an ecological context with a circulating lytic phage. We find that such endemic virus environments are often invasible by temperate strategies (i.e. $R_{\text{inv}}>1$) even when prophage impose a cost to cellular growth. This invasibility arises because lytic infections decrease susceptible host cell densities, and indirectly reduce niche competition between uninfected cells and a subpopulation of lysogens. Hence, our results provide direct support for the low host cell density adaptation hypothesis. However, our results also go further. We also evaluated the sensitivity of model findings to variation in super-infection exclusion. We find that when temperate phage impose a cost to cellular growth but confer immunity to lysogens against infection by lytic viruses, then temperate phage can invade in ecological contexts with pre-existing viruses where they would otherwise not be able to invade in a virus-free case. Hence, lytic viruses may actually enable the invasion of a broader range of temperate strategies. This finding provides additional mechanistic support consistent with studies of the evolution of viral strategies given long-term viral–host feedback (Berngruber et al. 2013; Wahl et al. 2018; Gulbudak and Weitz 2019). Note that such invasions are a first-step toward understanding long-term evolution. Indeed, subsequent invasions of an endemically infected state by temperate or purely lytic phage could lead to multi-strain coexistence—further complicating the benefits of being temperate.

While our study identifies possible conditions under which latency may evolve in phage–bacteria communities, additional theory is needed that explicitly models the long-term evolution of transmission strategies in viruses. In doing so, there are important scaffolds on which to build. For example, recent work (Wahl et al. 2018) used an adaptive dynamics framework which assumes mutations of small effect to consider how ecological context modulated the long-term evolution of a single trait related to temperate phage: the probability of integration. The researchers found that increased coupling between phage and host (e.g. the absence of an external supply of new hosts) increased the evolutionarily stable integration rate. Here, our work considers two phage traits related to the temperate lifestyle: the integration probability and the induction rate. Extending the present analysis to long-term evolution would therefore require consideration of the evolution of multiple traits. In doing so, we expect new complications to arise.

For example, our analysis assumes that resources affect cell growth but not viral fitness. However, viral life history traits can, in fact, depend on resource levels and host physiological state (Rabinovitch et al. 1999; Clasen and Elser 2007). Hence, future research should connect these microscopic traits with population level models. In addition, infected cell fate is strongly influenced by the cellular multiplicity of infection (cMOI), that is the number of co-infecting phage genomes in an individual cell. For phage *λ*, the fraction of lysogeny increases with increasing cMOI (Kourilsky 1973, 1975; Kourilsky and Knapp 1975). The mechanistic basis for this change has focused on feedback in the cell fate determination circuit (Weitz et al. 2008; Zeng et al. 2010; Golding 2016). From a game-theory point of view, phage may be balancing their risk of extinction in fluctuating environments (Avlund et al. 2009). The comprehensive understanding of how such features have evolved from an eco-evolutionary perspective is still needed. Moving forward, the integration of resource dynamics, multiple infections, and fluctuating ecological dynamics (Maslov and Sneppen 2015) are critical to a comprehensive understanding of why phage are temperate (and even why evolved temperate strategies are intermediate and/or responsive). In doing so, it will also be important to recognize that what may be evolutionary ‘optimal’ in a single-host environment will likely differ in a complex community when multiple hosts and viruses interact (Weitz 2015). For example, a lysogen may not necessarily confer immunity to co-circulating viral types, providing potential advantages to lytic phage in complex communities relative to those in single host–virus populations.

In closing, the loop-based analysis developed here provides a unified and tractable interpretation of temperate phage invasion fitness. Lysogeny provides a direct fitness benefit to viruses when hosts are rare (but resources are available) and also enables viruses to invade environments in which lytic viruses have reduced host densities and by extension niche competition. We speculate that a loop-based approach to measuring invasion fitness may be of service in the analysis of other parasite–host systems. Altogether, our results provide a principled framework for connecting intra-cellular exploitation of bacteria by phage with population and evolutionary-level outcomes. We hope this framework can facilitate analysis of the experimental evolution of latency in model phage–bacteria systems as well as shed light on drivers of variation in lysogeny in the environment.

## 4. Methods

### 4.1 Main models

We represent the models in Fig. 1 in terms of systems of ODEs. Note that the nonlinear population models with explicit infections (both resource-implicit and -explicit) are presented in Section 2.

The resource-implicit model with implicit infections given by Berngruber et al. (2013) is

(14) $$\begin{array}{ll} \dot{S} & =\overset{\text{logistic growth}}{\overset{︷}{r_{S}S\left(1-\frac{N}{K}\right)}}+\overset{\text{lysogens fail to transmit}}{\overset{︷}{r_{L}(1-\delta )L\left(1-\frac{N}{K}\right)}}-\overset{\text{infection}}{\overset{︷}{b\phi SV}}-\overset{\text{decay}}{\overset{︷}{mS}}\\ \dot{L} & =\overset{\text{logistic growth}}{\overset{︷}{r_{L}\delta L\left(1-\frac{N}{K}\right)}}+\overset{\text{lysogenic path}}{\overset{︷}{pb\phi SV}}-\overset{\text{induction}}{\overset{︷}{\gamma L}}-\overset{\text{decay}}{\overset{︷}{mL}}\\ \dot{V} & =\overset{\text{induction}}{\overset{︷}{\beta \gamma L}}+\overset{\text{lytic path}}{\overset{︷}{(1-p)\beta b\phi SV}}-\overset{\text{absorption}}{\overset{︷}{\phi NV}}-\overset{\text{decay}}{\overset{︷}{mV}} \end{array},$$

where N=S+L is the density of total cells, *L* is the density of infected cells, the susceptible cells density is *S*, and the free-virus density is *V*. More details can be found in Berngruber et al. (2013).

The resource-explicit model with implicit infections given by Stewart and Levin (1984) is

(15) $$\begin{array}{ll} \dot{R} & =\overset{\text{media inflow}}{\overset{︷}{\rho C}}-\overset{\text{nutrient consumption}}{\overset{︷}{e\psi (R)\left(L+(1-\alpha_{s})S\right)}}-\overset{\text{outflow}}{\overset{︷}{\rho R}}\\ \dot{S} & =\overset{\text{growth with fitness selection}}{\overset{︷}{(1-\alpha_{s})\psi (R)S}}-\overset{\text{infection}}{\overset{︷}{\phi SV}}+\overset{\text{segregation}}{\overset{︷}{\nu L}}-\overset{\text{outflow}}{\overset{︷}{\rho S}}\\ \dot{L} & =\overset{\text{growth}}{\overset{︷}{\psi (R)L}}+\overset{\text{lysogenic infection}}{\overset{︷}{p\phi SV}}-\overset{\text{induction}}{\overset{︷}{\gamma L}}-\overset{\text{segregation}}{\overset{︷}{\nu L}}-\overset{\text{outflow}}{\overset{︷}{\rho L}}\\ \dot{V} & =\overset{\text{induction}}{\overset{︷}{\beta \gamma L}}+\overset{\text{lytic infection}}{\overset{︷}{\beta (1-p)\phi SV}}-\overset{\text{absorption}}{\overset{︷}{\phi LV}}-\overset{\text{outflow}}{\overset{︷}{\rho V}} \end{array},$$

where *R*, *S*, *L*, and *V* denote the densities of resources, susceptible cells, lysogens, and virus particles, respectively. This model describes the dynamics of microbial populations in the chemostat, *ρ* is the inflow (and outflow) rate; additional model details in Stewart and Levin (1984).

### 4.2 Viral invasion analysis

The $R_{0}$ calculations for each model variant closely follow the procedures given by Diekmann, Heesterbeek, and Roberts (2010); complete calculations are provided in Supplementary Appendix B. To demonstrate the biological interpretation of NGM associated with temperate phage invasion dynamics, we present the NGM, Φ, of system of Equation (1) in Equation (5). The loop-based $R_{0}$ interpretation is inspired by Levins’ loop analysis (Levins 1974). For each model, we first compute $R_{0}$ via Equation (9) by an NGM approach, then, reformulate it into Equation (13) by identifying all the one- and two-generation loops. The loop-based results are summarized in Table 1 and the detailed calculations are given in Supplementary Appendix B.

### 4.3 Endemic invasion analysis

For each temperate phage model, we construct the mutant-resident system, and compute the invasion fitness of mutant viral strains ($R_{\text{inv}}$) at the resident endemic equilibrium via an NGM approach (Hurford, Cownden, and Day 2010; Wahl et al. 2018); see Supplementary Appendix D.

## Supplementary Material

veaa042_Supplementary_Data
