# Supplementary material for: When to be temperate: on the fitness benefits of lysis vs. lysogeny
Source: Virus Evol. 2020 May 22;6(2):veaa042. doi: 10.1093/ve/veaa042 (PMC9532926; doi:10.1093/ve/veaa042)
Supplement: veaa042_Supplementary_Data [file ve_6_2_veaa042_s1.pdf]

Supplementary Information: When to be Temperate: On the Fitness Benefits of Lysis vs. Lysogeny

Guanlin Li[1, 2], Michael H. Cortez[3], Jonathan Dushoff[4,5,6], and Joshua S. Weitz *[2,7]

[1]Interdisciplinary Graduate Program in Quantitative Biosciences, Georgia Institute of Technology, Atlanta, GA, 30332, USA
[2]School of Physics, Georgia Institute of Technology, Atlanta, GA, 30332, USA
[3]Department of Biological Science, Florida State University, Tallahassee, FL 32306, USA
[4]Department of Biology, McMaster University, Hamilton, ON, L8S 4L8, Canada
[5]Department of Mathematics and Statistics, McMaster University, Hamilton, ON, L8S 4L8, Canada
[6]M. G. DeGroote Institute for Infectious Disease Research, McMaster University, Hamilton, ON, L8S 4L8, Canada
[7]School of Biological Sciences, Georgia Institute of Technology, Atlanta, GA, 30332 USA

May 15, 2020

A Nonlinear, Population Model of Temperate Phage Dynamics

A.1 Main models

The resource-implicit model with explicit infections from the main text is

$$
\begin{aligned}
\dot{S} &= \overbrace{r_S S\left(1-\frac{N}{K}\right)}^{\text{logistic growth}} - \overbrace{\phi S V}^{\text{infection}} - \overbrace{d_S S}^{\text{decay}} \\
\dot{E} &= \overbrace{\phi S V}^{\text{infection}} - \overbrace{\lambda E}^{\text{transition}} - \overbrace{d_E E}^{\text{decay}} \\
\dot{L} &= \overbrace{p\lambda E}^{\text{lysogenic infection}} + \overbrace{r_L L\left(1-\frac{N}{K}\right)}^{\text{logistic growth}} - \overbrace{\gamma L}^{\text{induction}} - \overbrace{d_L L}^{\text{decay}} \\
\dot{I} &= \overbrace{(1-p)\lambda E}^{\text{lytic infection}} + \overbrace{\gamma L}^{\text{induction}} - \overbrace{\eta I}^{\text{lysis}} - \overbrace{d_I I}^{\text{decay}} \\
\dot{V} &= \overbrace{\beta\eta I}^{\text{burst}} - \overbrace{\phi N V}^{\text{infection}} - \overbrace{m V}^{\text{decay}}
\end{aligned}
\tag{A.1}
$$

where S, E, L, I and V denote the densities of susceptible cells, exposed infected cells, lysogens, lytic-fated infected cells and virus particles respectively, and $N = S + E + L + I$ is the total cell density. Parameters r_S and r_L denote the maximal cellular growth rates of susceptible cells and lysogens, K is the carrying capacity, ϕ is the adsorption rate, d_S, d_E, d_L and d_I are the cellular death rates of susceptible cells, exposed infected cells, lysogens and lytic-fated infected cells respectively, λ is the transition rate from exposed cells to the fate determined cells, p is the probability of lysogenization, γ is the induction

*Electronic address: jsweitz@gatech.edu ; URL: http://ecotheory.biology.gatech.edu

rate, η is the lysis rate, β is the burst size and m is the virion decay rate. The resource-explicit model with explicit infections from the main text is

$$\begin{aligned}
\dot{R} &= \overbrace{J}^{\text{influx}} - \overbrace{e\psi(R)\left(L+(1-\alpha_s)S\right)}^{\text{cumulative uptake of nutrients by cells}} - \overbrace{d_R R}^{\text{decay}} \\
\dot{S} &= \overbrace{(1-\alpha_s)\psi(R)S}^{\text{growth with fitness selection}} - \overbrace{\phi SV}^{\text{infection}} - \overbrace{d_S S}^{\text{decay}} \\
\dot{E} &= \overbrace{\phi SV}^{\text{infection}} - \overbrace{\lambda E}^{\text{transition}} - \overbrace{d_E E}^{\text{decay}} \\
\dot{L} &= \overbrace{p\lambda E}^{\text{lysogenic infection}} + \overbrace{\psi(R)L}^{\text{lysogens growth}} - \overbrace{\gamma L}^{\text{induction}} - \overbrace{d_L L}^{\text{decay}} \\
\dot{I} &= \overbrace{(1-p)\lambda E}^{\text{lytic infection}} + \overbrace{\gamma L}^{\text{induction}} - \overbrace{\eta I}^{\text{lysis}} - \overbrace{d_I I}^{\text{decay}} \\
\dot{V} &= \overbrace{\beta\eta I}^{\text{burst}} - \overbrace{\phi NV}^{\text{infection}} - \overbrace{mV}^{\text{decay}}
\end{aligned} \tag{A.2}$$

where R, S, E, L, I and V denote the densities of resources, susceptible cells, exposed infected cells, lysogens, lytic-fated infected cells and virus particles, respectively, and $N = S + E + L + I$ is the total cell density. The growth function $\psi(R) = \mu_{max}R/(R_{in} + R)$ is a Monod equation, where μ_{max} is the maximal cellular growth rate and R_{in} is the half-saturation constant. The parameters J and d_R are the influx and decay rates of resources, e is the host conversion efficiency, α_S is the selection coefficient that measures the relative fitness between lysogens and susceptible cells. All other parameters are defined as in model [A.1]. The resource-implicit model with implicit infections in Berngruber et al. (2013) is

$$\begin{aligned}
\dot{S} &= \overbrace{r_S S\left(1-\frac{N}{K}\right)}^{\text{logistic growth}} + \overbrace{r_L(1-\delta)L\left(1-\frac{N}{K}\right)}^{\text{lysogens fail to vertically transmit}} - \overbrace{b\phi SV}^{\text{infection}} - \overbrace{mS}^{\text{decay}} \\
\dot{L} &= \overbrace{r_L\delta L\left(1-\frac{N}{K}\right)}^{\text{logistic growth}} + \overbrace{pb\phi SV}^{\text{lysogenic path}} - \overbrace{\gamma L}^{\text{induction}} - \overbrace{mL}^{\text{decay}} \\
\dot{V} &= \overbrace{\beta\gamma L}^{\text{induction}} + \overbrace{(1-p)\beta b\phi SV}^{\text{lytic path}} - \overbrace{\phi NV}^{\text{absorption to cells}} - \overbrace{mV}^{\text{decay}}
\end{aligned} \tag{A.3}$$

where $N = S + L$ is the density of total cells, L is the density of infected cells, the density of susceptible cells is S and the free-virus density is V. More details about model [A.3] can be found in (Berngruber et al. (2013)). The resource-explicit model with implicit infections from Stewart and Levin (1984) is

$$\begin{aligned}
\dot{R} &= \overbrace{\rho C}^{\text{media inflow}} - \overbrace{e\psi(R)\left(L+(1-\alpha_s)S\right)}^{\text{nutrient consumption}} - \overbrace{\rho R}^{\text{outflow}} \\
\dot{S} &= \overbrace{(1-\alpha_s)\psi(R)S}^{\text{growth with fitness selection}} - \overbrace{\phi SV}^{\text{infection}} + \overbrace{\nu L}^{\text{vegetative segregation from lysogens}} - \overbrace{\rho S}^{\text{outflow}} \\
\dot{L} &= \overbrace{\psi(R)L}^{\text{lysogens growth}} + \overbrace{p\phi SV}^{\text{lysogenic infection}} - \overbrace{\gamma L}^{\text{induction}} - \overbrace{\nu L}^{\text{vegetative segregation}} - \overbrace{\rho L}^{\text{outflow}} \\
\dot{V} &= \overbrace{\beta\gamma L}^{\text{induction}} + \overbrace{\beta(1-p)\phi SV}^{\text{lytic infection}} - \overbrace{\phi LV}^{\text{absorption to lysogens}} - \overbrace{\rho V}^{\text{outflow}}
\end{aligned} \tag{A.4}$$

where R, S, L, and V denote the densities of resources, susceptible cells, lysogens and virus particles, respectively. Model [A.4] describes the dynamics of populations in a chemostat, where ρ is the inflow (and outflow) rate and ν is the segregation rate whereby lysogens become susceptible cells. See (Stewart and Levin (1984)) for more details.

In the four model variants, the phage strategies are defined by two parameters: p defines the probability a virus enters the lysogenic pathway and γ defines the induction rate after a virus enters the lysogenic pathway. We define the viral strategy space Θ as

$$\Theta \;=\; \{(p,\gamma) : 0 \le p \le 1,\ \gamma_{min} \le \gamma \le \gamma_{max}\} \tag{A.5}$$

where $\gamma_{min} > 0$.

A.2 From explicit infections to implicit infections

In this section, we show that the explicit infection models [A.1] and [A.2] can be reduced to the models with implicit infections via a quasi–steady–state (QSS) approximation.

For model [A.1] and model [A.2], we assume the (lysis-lysogeny decision) transition process and lysis process are extremely rapid in comparison to all the other biological processes. In other words, we let $\lambda \gg d_E$ and $\eta \gg d_I$. The population dynamics of exposed cells from model [A.1] and model [A.2] can be rewritten as

$$\epsilon \dot{E} = \frac{\phi SV}{\lambda} - E(1 + \epsilon d_E) \approx \frac{\phi SV}{\lambda} - E \tag{A.6}$$

where $\epsilon = 1/\lambda \ll 1$. Hence, the QSS equilibrium density of exposed cells population is $E^\epsilon = \phi SV/\lambda$. Using E^ϵ, the population dynamics of lytic-fated infected cells from model [A.1] and model [A.2] are rewritten as

$$\epsilon' \dot{I} = \frac{(1-p)\lambda E^\epsilon + \gamma L}{\eta} - I(1 + \epsilon' d_I) \approx \frac{(1-p)\phi SV + \gamma L}{\eta} - I \tag{A.7}$$

where $\epsilon' = 1/\eta \ll 1$. Hence, the QSS equilibrium density of lytic-fated infected cells is $I^{\epsilon'} = [(1 - p)\phi SV + \gamma L]/\eta$. Substituting E^ϵ and $I^{\epsilon'}$ into model [A.1] reduces it to the S, L, V-system with implicit infections,

$$\begin{aligned} \dot{S} &= r_S S \left(1 - \frac{N}{K}\right) - \phi SV - d_S S \\ \dot{L} &= p\phi SV + r_L L \left(1 - \frac{N}{K}\right) - \gamma L - d_L L \\ \dot{V} &= \beta(1-p)\phi SV + \beta\gamma L - \phi NV - mV. \end{aligned} \tag{A.8}$$

Substituting E^ϵ and $I^{\epsilon'}$ into model [A.2] reduces it to the R, S, L, V-system with implicit infections,

$$\begin{aligned} \dot{R} &= J - e\psi(R)\left(L + (1-\alpha_s)S\right) - d_R R \\ \dot{S} &= (1-\alpha_s)\psi(R)S - \phi SV - d_S S \\ \dot{L} &= p\phi SV + \psi(R)L - \gamma L - d_L L \\ \dot{V} &= \beta(1-p)\phi SV + \beta\gamma L - \phi NV - mV. \end{aligned} \tag{A.9}$$

B Viral Invasion Analysis

B.1 Virus-free equilibrium

For the resource-implicit models [A.1] and [A.3], there are only susceptible cells (S^*) in the virus-free environments. The virus-free equilibrium of model [A.1] is

$$(S^*, 0, 0, 0, 0), \quad \text{where} \quad S^* = K\left(1 - \frac{d_S}{r_S}\right). \tag{B.10}$$

The virus-free equilibrium of model [A.3] is

$$(S^*, 0, 0), \quad \text{where} \quad S^* = K\left(1 - \frac{d_S}{m}\right). \tag{B.11}$$

For the resource-explicit models [A.2] and [A.4], there are both resources and susceptible cells (R^*, S^*) in the virus-free environments. The virus-free equilibrium of model [A.2] is

$$(R^*, S^*, 0, 0, 0, 0), \quad \text{where} \quad R^* = \frac{R_{in} d_S}{(1-\alpha_s)\mu_{max} - d_S} \quad \text{and} \quad S^* = \frac{J - d_R R^*}{e d_S}. \tag{B.12}$$

The virus-free equilibrium of model [A.4] is

$$(R^*, S^*, 0, 0), \quad \text{where} \quad R^* = \frac{R_{in}\rho}{(1-\alpha_s)\mu_{max} - \rho} \quad \text{and} \quad S^* = \frac{C - R^*}{e}. \tag{B.13}$$

B.2 Next-generation matrix approach

We start by computing the next-generation matrix (NGM) for the resource implicit model with explicit infections [A.1]. Consider the Jacobian of the model [A.1] evaluated at the virus-free equilibrium, Eq. [B.12]. We denote $\mathcal{J}$ as the submatrix of Jacobian for the E, L, I, V-subsystem. We decompose the submatrix as $\mathcal{J} = \mathcal{F} + \mathcal{V}$ where the transmission matrix $\mathcal{F}$ and the transition matrix $\mathcal{V}$ are

$$\mathcal{F} = \begin{bmatrix} 0 & 0 & 0 & \phi S^* \\ 0 & r_L(1-\frac{S^*}{K}) & 0 & 0 \\ 0 & 0 & 0 & 0 \\ 0 & 0 & 0 & 0 \end{bmatrix}, \quad \mathcal{V} = \begin{bmatrix} -(\lambda + d_E) & 0 & 0 & 0 \\ p\lambda & -(\gamma + d_L) & 0 & 0 \\ (1-p)\lambda & \gamma & -(\eta + d_I) & 0 \\ 0 & 0 & \beta\eta & -(\phi S^* + m) \end{bmatrix}.$$

Via NGM theory (Diekmann et al. (2010)), the basic reproduction number $\mathcal{R}_0$ corresponds to the largest eigenvalue of the matrix $-\mathcal{F}\mathcal{V}^{-1}$, namely $\mathcal{R}_0 = \varrho(-\mathcal{F}\mathcal{V}^{-1})$ where $\varrho(M)$ is the spectral radius of the matrix M. There are two epidemiological birth states in model [A.1], hence, there are only two non-zero eigenvalues in $-\mathcal{F}\mathcal{V}^{-1}$. Here we introduce an augmented operator Q, where Q is a matrix in $\mathbb{R}^{4\times 2}$ with unit vectors in columns 1 and 2. The spectral radius of the 4×4 matrix $-\mathcal{F}\mathcal{V}^{-1}$ is the same as the spectral radius of the 2×2 matrix $-Q^T\mathcal{F}\mathcal{V}^{-1}Q$. For convenience, we define the next generation matrix of model [A.1] as

$$\Phi = -Q^T\mathcal{F}\mathcal{V}^{-1}Q = \begin{bmatrix} \Phi_{11} & \Phi_{12} \\ \Phi_{21} & \Phi_{22} \end{bmatrix},$$

where the entries of Φ are

$$\begin{aligned}
\Phi_{11} &= \overbrace{\left(\frac{p\lambda}{\lambda + d_E}\right)}^{E\to L}\overbrace{\left(\frac{\gamma}{\gamma + d_L}\right)}^{L\to I}\overbrace{\left(\frac{\beta\,\eta}{\eta + d_I}\right)}^{I\to V}\overbrace{\left(\frac{\phi S^*}{\phi S^* + m}\right)}^{V\to E} + \overbrace{\left(\frac{(1-p)\lambda}{\lambda + d_E}\right)}^{E\to I}\overbrace{\left(\frac{\beta\,\eta}{\eta + d_I}\right)}^{I\to V}\overbrace{\left(\frac{\phi S^*}{\phi S^* + m}\right)}^{V\to E} \\
\Phi_{22} &= \frac{\overbrace{r_L(1 - S^*/K)}^{L\to L}}{\gamma + d_L} \\
\Phi_{12} &= \overbrace{\left(\frac{\gamma}{\gamma + d_L}\right)}^{L\to I}\overbrace{\left(\frac{\beta\,\eta}{\eta + d_I}\right)}^{I\to V}\overbrace{\left(\frac{\phi S^*}{\phi S^* + m}\right)}^{V\to E} \\
\Phi_{21} &= \overbrace{\left(\frac{p\lambda}{\lambda + d_E}\right)}^{E\to L}\overbrace{\frac{r_L(1 - S^*/K)}{\gamma + d_L}}^{L\to L}.
\end{aligned} \tag{B.14}$$

Entry Φ_{ij} represents the expected number of new infected individuals in state i, generated by one infected individual at state j $(i, j = L, E)$, accounting for new infections that arise via the lytic and lysogenic pathways. Φ can be rewritten in terms of the basic reproductive number for viruses with purely lytic strategies ($p = 0$; $\mathcal{R}^{hor}$) and the basic reproductive number for viruses with purely lysogenic strategies ($p = 1, \gamma = 0$; $\mathcal{R}^{ver}$),

$$\Phi = \begin{bmatrix} \mathcal{R}^{hor}\widetilde{\gamma}p + (1-p)\mathcal{R}^{hor} & \mathcal{R}^{hor}(\widetilde{\gamma}/\widetilde{\lambda}) \\ \mathcal{R}^{ver}p\widetilde{\lambda}(1-\widetilde{\gamma}) & \mathcal{R}^{ver}(1-\widetilde{\gamma}) \end{bmatrix} \tag{B.15}$$

where

$$\widetilde{\gamma} = \frac{\gamma}{\gamma + d_L}, \quad \widetilde{\lambda} = \frac{\lambda}{\lambda + d_E}, \quad \mathcal{R}^{hor}(S^*) = \frac{\beta\eta\phi S^*\lambda}{(\eta + d_I)(\phi S^* + m)(\lambda + d_E)}, \quad \mathcal{R}^{ver}(S^*) = \frac{r_L}{d_L}\left(1 - \frac{S^*}{K}\right). \tag{B.16}$$

Next we compute the NGM for the implicit model with implicit infections [A.3]. We decompose the linearized infected subsystem of model [A.3], at the virus-free equilibrium, Eq. [B.11], into the transmission matrix $\mathcal{F}$ and the transition matrix $\mathcal{V}$:

$$\mathcal{F} = \begin{bmatrix} r_L\delta\left(1 - \frac{S^*}{K}\right) & bp\phi S^* \\ 0 & (1-p)\beta b\phi S^* \end{bmatrix}, \quad \mathcal{V} = \begin{bmatrix} -(\gamma + m) & 0 \\ \gamma\beta & -(m + \phi S^*) \end{bmatrix}.$$

The NGM of model [A.3] is defined as

$$\Phi = -\mathcal{F}\mathcal{V}^{-1} = \begin{bmatrix} \overbrace{\frac{r_L\delta\left(1-\frac{S^*}{K}\right)}{\gamma+m}}^{L\to L} + \overbrace{\frac{\beta\gamma}{\gamma+m}}^{L\to V}\overbrace{\frac{pb\phi S^*}{(\phi S^*+m)}}^{V\to L} & \overbrace{pb\frac{\phi S^*}{\phi S^*+m}}^{V\to L} \\ \underbrace{\frac{\beta\gamma}{\gamma+m}}_{}^{L\to V}\overbrace{(1-p)b\beta\frac{\phi S^*}{\phi S^*+m}}^{V\to V} & \overbrace{(1-p)b\beta\frac{\phi S^*}{\phi S^*+m}}^{V\to V} \end{bmatrix}. \tag{B.17}$$

As before, the NGM can be rewritten as

$$\Phi = \begin{bmatrix} \mathcal{R}^{hor}\widetilde{\gamma}p + (1-\widetilde{\gamma})\mathcal{R}^{ver} & \mathcal{R}^{hor}(p/\beta) \\ \mathcal{R}^{hor}(1-p)\widetilde{\gamma}\beta & \mathcal{R}^{hor}(1-p) \end{bmatrix} \tag{B.18}$$

where

$$\widetilde{\gamma} = \frac{\gamma}{\gamma+m}\ , \quad \mathcal{R}^{hor}(S^*) = \frac{b\beta\phi S^*}{\phi S^*+m}\ , \quad \mathcal{R}^{ver}(S^*) = \frac{r_L\delta}{m}\left(1-\frac{S^*}{K}\right). \tag{B.19}$$

Similarly, we compute the NGM for the resource explicit model with explicit infections [A.2]. We decompose the linearized infected subsystem of model [A.2] evaluated at the virus-free equilibrium, Eq. [B.12], into the transmission matrix $\mathcal{F}$ and transition matrix $\mathcal{V}$:

$$\mathcal{F} = \begin{bmatrix} 0 & 0 & 0 & \phi S^* \\ 0 & \psi(R^*) & 0 & 0 \\ 0 & 0 & 0 & 0 \\ 0 & 0 & 0 & 0 \end{bmatrix}\ , \quad \mathcal{V} = \begin{bmatrix} -(\lambda+d_E) & 0 & 0 & 0 \\ p\lambda & -(\gamma+d_L) & 0 & 0 \\ (1-p)\lambda & \gamma & -(\eta+d_I) & 0 \\ 0 & 0 & \beta\eta & -(\phi S^*+m) \end{bmatrix}.$$

The NGM of model [A.2] is defined as

$$\Phi = -Q^T\mathcal{F}\mathcal{V}^{-1}Q = \begin{bmatrix} \Phi_{11} & \Phi_{12} \\ \Phi_{21} & \Phi_{22} \end{bmatrix},$$

where Q is a matrix in $\mathbb{R}^{4\times 2}$ with unit vectors in columns 1 and 2. The entries of Φ are

$$\begin{aligned}
\Phi_{11} &= \overbrace{\left(\frac{p\lambda}{\lambda+d_E}\right)}^{E\to L}\overbrace{\left(\frac{\gamma}{\gamma+d_L}\right)}^{L\to I}\overbrace{\left(\frac{\beta\,\eta}{\eta+d_I}\right)}^{I\to V}\overbrace{\left(\frac{\phi S^*}{\phi S^*+m}\right)}^{V\to E} + \overbrace{\left(\frac{(1-p)\lambda}{\lambda+d_E}\right)}^{E\to I}\overbrace{\left(\frac{\beta\,\eta}{\eta+d_I}\right)}^{I\to V}\overbrace{\left(\frac{\phi S^*}{\phi S^*+m}\right)}^{V\to E} \\
\Phi_{22} &= \overbrace{\frac{\psi(R^*)}{\gamma+d_L}}^{L\to L} \\
\Phi_{12} &= \overbrace{\left(\frac{\gamma}{\gamma+d_L}\right)}^{L\to I}\overbrace{\left(\frac{\beta\,\eta}{\eta+d_I}\right)}^{I\to V}\overbrace{\left(\frac{\phi S^*}{\phi S^*+m}\right)}^{V\to E} \\
\Phi_{21} &= \overbrace{\left(\frac{p\lambda}{\lambda+d_E}\right)}^{E\to L}\overbrace{\frac{\psi(R^*)}{\gamma+d_L}}^{L\to L}.
\end{aligned} \tag{B.20}$$

As before, we can rewrite the NGM as

$$\Phi = \begin{bmatrix} \mathcal{R}^{hor}\widetilde{\gamma}p + (1-p)\mathcal{R}^{hor} & \mathcal{R}^{hor}(\widetilde{\gamma}/\widetilde{\lambda}) \\ \mathcal{R}^{ver}p\widetilde{\lambda}(1-\widetilde{\gamma}) & \mathcal{R}^{ver}(1-\widetilde{\gamma}) \end{bmatrix} \tag{B.21}$$

where

$$\widetilde{\gamma} = \frac{\gamma}{\gamma+d_L}\ , \quad \widetilde{\lambda} = \frac{\lambda}{\lambda+d_E}\ , \quad \mathcal{R}^{hor}(S^*) = \frac{\beta\eta\phi S^*\lambda}{(\eta+d_I)(\phi S^*+m)(\lambda+d_E)}\ , \quad \mathcal{R}^{ver}(R^*) = \frac{\psi(R^*)}{d_L}. \tag{B.22}$$

Finally, we compute the NGM for the resource explicit model with implicit infections [A.4]. We decompose the linearized infected subsystem of model [A.4], at the virus-free equilibrium, Eq. [B.13], in to the transmission matrix $\mathcal{F}$ and the transition matrix $\mathcal{V}$:

$$\mathcal{F} = \begin{bmatrix} \psi(R^*) & p\phi S^* \\ 0 & (1-p)\beta\phi S^* \end{bmatrix} , \quad \mathcal{V} = \begin{bmatrix} -(\rho+\gamma+\nu) & 0 \\ \gamma\beta & -\rho \end{bmatrix}.$$

The NGM of model [A.4] is defined as

$$\Phi = -\mathcal{F}\mathcal{V}^{-1} = \begin{bmatrix} \overbrace{\frac{\psi(R^*)}{\rho+\gamma+\nu}}^{L\to L} + \overbrace{\frac{\beta\gamma}{\rho+\gamma+\nu}}^{L\to V} \overbrace{p\frac{\phi S^*}{\rho}}^{V\to L} & \overbrace{p\frac{\phi S^*}{\rho}}^{V\to L} \\ \underbrace{\frac{\beta\gamma}{\rho+\gamma+\nu}}_{}^{L\to V} \overbrace{(1-p)\frac{\beta\phi S^*}{\rho}}^{V\to V} & \overbrace{(1-p)\frac{\beta\phi S^*}{\rho}}^{V\to V} \end{bmatrix}. \tag{B.23}$$

As before, we can rewrite the NGM as

$$\Phi = \begin{bmatrix} \mathcal{R}^{hor}\widetilde{\gamma}p + (1-\widetilde{\gamma})\mathcal{R}^{ver} & \mathcal{R}^{hor}(p/\beta) \\ \mathcal{R}^{hor}(1-p)\widetilde{\gamma}\beta & \mathcal{R}^{hor}(1-p) \end{bmatrix} \tag{B.24}$$

where

$$\widetilde{\gamma} = \frac{\gamma}{\gamma+\rho+\nu} , \quad \mathcal{R}^{hor}(S^*) = \frac{\phi S^*\beta}{\rho} , \quad \mathcal{R}^{ver}(R^*) = \frac{\psi(R^*)}{\rho+\nu}. \tag{B.25}$$

Notably, the next-generation matrices for all four model variants are 2×2 matrices, as shown in Eq. [B.15], Eq. [B.17], Eq. [B.21] and Eq. [B.24]. The traces, $T(\Phi)$, and determinants, $D(\Phi)$, of each next-generation matrix can be written as

$$T(\Phi) = \mathcal{R}^{hor}\widetilde{\gamma}p + (1-p)\mathcal{R}^{hor} + (1-\widetilde{\gamma})\mathcal{R}^{ver} , \quad D(\Phi) = \mathcal{R}^{hor}\mathcal{R}^{ver}(1-p)(1-\widetilde{\gamma}). \tag{B.26}$$

where the values of $\mathcal{R}^{hor}, \mathcal{R}^{ver}$ and $\widetilde{\gamma}$ are given in Eq. [B.16], Eq. [B.19], Eq. [B.22] and Eq. [B.25]. The dominate eigenvalue $\mathcal{R}_0$ can be obtained from the trace and the determinant of the next-generation matrix,

$$\mathcal{R}_0 = \frac{1}{2}\left(T(\Phi) + \sqrt{T(\Phi)^2 - 4D(\Phi)}\right). \tag{B.27}$$

In general, $\mathcal{R}_0 = \max(\mathcal{R}^{hor}, (1-\widetilde{\gamma})\mathcal{R}^{ver})$ when $p = 0$. However, we define $\mathcal{R}_0 = \mathcal{R}^{hor}$ when $p = 0$ because that matches the biological scenario defined by a virus with a purely lytic strategy.

B.3 Loop-based interpretation of $\mathcal{R}_0$

Motivated by Levins' loop analysis (Levins (1974)), we can rewrite Eq. [B.27] in a biological interpretable way. We define, $P_1 = (1-p)\mathcal{R}^{hor}$, $P_2 = (1-\widetilde{\gamma})\mathcal{R}^{ver}$ and $P_3 = p\widetilde{\gamma}\mathcal{R}^{hor}$. Then, Eq. [B.27] can be written as

$$2\mathcal{R}_0 = P_1 + P_2 + P_3 + \sqrt{P_1^2 + P_2^2 + P_3^2 + 2P_1P_3 + 2P_2P_3 - 2P_1P_2}. \tag{B.28}$$

A full description of this interpretation is presented in the main text.

C Maximization of $\mathcal{R}_0$ and Feasible Invasion Strategies

C.1 Preliminaries

A strategy (p^*, γ^*) maximizes the basic reproduction number corresponding to the viral invasion fitness as measured at the individual level if

$$\mathcal{R}_0(p^*, \gamma^*) \geq \mathcal{R}_0(p, \gamma) \quad \forall\ (p, \gamma) \in \Theta.$$

Given its use in the calculations and proofs, we note that while $\widetilde{\gamma} = \gamma/(\gamma + d_L)$ lies in the closed interval

$$\widetilde{\gamma} \in \left[\frac{\gamma_{min}}{\gamma_{min}+d_L} , \frac{\gamma_{max}}{\gamma_{max}+d_L}\right] \quad \text{where} \quad 0 < \frac{\gamma_{min}}{\gamma_{min}+d_L} < \frac{\gamma_{max}}{\gamma_{max}+d_L} < 1,$$

we generally use $\widetilde{\gamma} \in (0,1)$ instead of $\gamma \in [\gamma_{min}, \gamma_{max}]$ for convenience.

Proposition C.1 *The basic reproduction number* $\mathcal{R}_0(p,\gamma)$*, Eq. [B.27], is marginal continuous on* $(p,\gamma) \in \Theta$ *and marginal differentiable on* $(p,\gamma) \in \Theta$ *except at* $\big(0,\ \big(\frac{\mathcal{R}^{ver}}{\mathcal{R}^{hor}} - 1\big)\, d_L\big)$.

Proof. The marginal continuity of $\mathcal{R}_0(p,\gamma)$ on $(p,\gamma) \in \Theta$ is obvious. Let $\theta \in \{p, \widetilde{\gamma}\}$, the derivative of $\mathcal{R}_0(p,\widetilde{\gamma})$ with respect to θ is

$$\frac{\partial \mathcal{R}_0}{\partial \theta} = \frac{1}{2}\left[T_\theta + \frac{T_\theta T - 2D_\theta}{\sqrt{T^2 - 4D}}\right]$$

where $T_\theta = \frac{\partial T}{\partial \theta}$ and $D_\theta = \frac{\partial D}{\partial \theta}$. Thus, we need $T^2 - 4D$ to be strictly positive to ensure the marginal differentiability. We write out $T^2 - 4D$ in terms of p and $\widetilde{\gamma}$

$$T^2 - 4D = \left[\mathcal{R}^{hor}(1-p) - \mathcal{R}^{ver}(1-\widetilde{\gamma})\right]^2 + 2\mathcal{R}^{hor}p\widetilde{\gamma}\left[\mathcal{R}^{hor}(1-p) + \mathcal{R}^{ver}(1-\widetilde{\gamma})\right] + (\mathcal{R}^{hor}p\widetilde{\gamma})^2 \ .$$

Algebraic manipulation shows that $T^2 - 4D > 0$ everywhere in Θ except at $\big(0,\ \big(\frac{\mathcal{R}^{ver}}{\mathcal{R}^{hor}} - 1\big)\, d_L\big)$. □

Lemma C.2 *Given a fixed* γ *satisfying* $0 < \gamma_{min} \le \gamma \le \gamma_{max}$*, the basic reproduction number* $\mathcal{R}_0(p,\gamma)$ *is uniquely maximized at* $p^* = 1$ *if* $\mathcal{R}^{hor} < \mathcal{R}^{ver}$*. In contrast, the basic reproduction number* $\mathcal{R}_0(p,\gamma)$ *is uniquely maximized at* $p^* = 0$ *if* $\mathcal{R}^{hor} > \mathcal{R}^{ver}$*.*

Proof. In the proof, we assume $\mathcal{R}^{hor} < \mathcal{R}^{ver}$; the case $\mathcal{R}^{hor} > \mathcal{R}^{ver}$ can be proved with a similar argument. The uniqueness of the maximal strategy p^* is showed by the strict monotonicity of $\mathcal{R}_0$ for all $p \in [0,1]$. Calculating the derivative of $\mathcal{R}_0$ with respect to p yields

$$\begin{aligned}
\frac{\partial \mathcal{R}_0}{\partial p} > 0 \quad &\Leftrightarrow \quad \frac{1}{2}\left[T_p + \frac{T_p T - 2D_p}{\sqrt{T^2 - 4D}}\right] \ > \ 0 \\
&\Leftrightarrow \quad \left[4D_p - 2T_p T\right]^2 \ > \ 4T_p^2\left[T^2 - 4D\right] \\
&\Leftrightarrow \quad T_p^2 D \ > \ D_p T_p T - D_p^2
\end{aligned}$$

where the derivatives are $T_p = \mathcal{R}^{hor}(\widetilde{\gamma} - 1) < 0$ and $D_p = -\mathcal{R}^{hor}\mathcal{R}^{ver}(1-\widetilde{\gamma}) < 0$. Note that

$$2T_pT - 4D_p = 2\mathcal{R}^{hor}(1-\widetilde{\gamma})\left[(\mathcal{R}^{ver} - \mathcal{R}^{hor}) + \mathcal{R}^{hor}p(1-\widetilde{\gamma}) + \widetilde{\gamma}(\mathcal{R}^{ver} - \mathcal{R}^{hor}p)\right] > 0 \ .$$

Substitution yields

$$\begin{aligned}
&T_p^2 D > D_p T_p T - D_p^2 \\
&\quad \Leftrightarrow (\mathcal{R}^{hor})^2\mathcal{R}^{ver}(\widetilde{\gamma} - 1)^2\left[\mathcal{R}^{hor}\mathcal{R}^{ver}(1-\widetilde{\gamma})(1-p) - \mathcal{R}^{ver}(\mathcal{R}^{hor}\widetilde{\gamma}p + \mathcal{R}^{hor}(1-p) + \mathcal{R}^{ver}(1-\widetilde{\gamma}) - \mathcal{R}^{ver})\right] > 0 \\
&\quad \Leftrightarrow (\mathcal{R}^{hor})^2\mathcal{R}^{ver}(\widetilde{\gamma} - 1)^2\widetilde{\gamma}(\mathcal{R}^{ver} - \mathcal{R}^{hor}) > 0 \\
&\quad \Leftrightarrow \mathcal{R}^{ver} - \mathcal{R}^{hor} > 0 \ .
\end{aligned}$$

Therefore, the basic reproduction number is a strictly monotone increasing function of p in the range of $[0,1]$. This implies $\mathcal{R}_0$ is maximized at $p^* = 1$ and minimized at $p = 0$ by the continuity of $\mathcal{R}_0$. □

Lemma C.3 *Given a fixed* p *satisfying* $0 < p \le 1$*, the basic reproduction number* $\mathcal{R}_0(p,\gamma)$ *is uniquely maximized at* $\gamma^* = \gamma_{min}$ *if* $\mathcal{R}^{hor} < \mathcal{R}^{ver}$*. In contrast, the basic reproduction number* $\mathcal{R}_0(p,\gamma)$ *is uniquely maximized at* $\gamma^* = \gamma_{max}$ *if* $\mathcal{R}^{hor} > \mathcal{R}^{ver}$*.*

Proof. The proof is similar to the proof of lemma (C.2). According to proposition (C.1), we fix $p \neq 0$ such that $\mathcal{R}_0$ is differentiable on $\gamma_{min} \le \gamma \le \gamma_{max}$. We assume $\mathcal{R}^{hor} < \mathcal{R}^{ver}$; the proof for $\mathcal{R}^{hor} > \mathcal{R}^{ver}$ is similar. Note by the chain rule that

$$\frac{\partial \mathcal{R}_0}{\partial \gamma} = \frac{\partial \mathcal{R}_0}{\partial \widetilde{\gamma}}\frac{\partial \widetilde{\gamma}}{\partial \gamma} \quad \text{where} \quad \frac{\partial \widetilde{\gamma}}{\partial \gamma} > 0 \ .$$

Let $\widetilde{\gamma} \in (0,1)$. Differentiating $\mathcal{R}_0$ yields

$$\frac{\partial \mathcal{R}_0}{\partial \widetilde{\gamma}} \ = \ \frac{1}{2}\left[T_{\widetilde{\gamma}} + \frac{T_{\widetilde{\gamma}}T - 2D_{\widetilde{\gamma}}}{\sqrt{T^2 - 4D}}\right].$$

Given $\mathcal{R}^{hor} < \mathcal{R}^{ver}$, the derivatives are $T_{\tilde{\gamma}} = \mathcal{R}^{hor}p - \mathcal{R}^{ver} < 0$ and $D_{\tilde{\gamma}} = -\mathcal{R}^{hor}\mathcal{R}^{ver}(1-p) < 0$. The sign of $T_{\tilde{\gamma}}T - 2D_{\tilde{\gamma}}$ is unknown, which can be expanded as

$$T_{\tilde{\gamma}}T - 2D_{\tilde{\gamma}} = \tilde{\gamma}(\mathcal{R}^{hor}p - \mathcal{R}^{ver})^2 + (\mathcal{R}^{hor})^2 p(1-p) + \mathcal{R}^{ver}(\mathcal{R}^{hor} - \mathcal{R}^{ver}) \quad .$$

If $T_{\tilde{\gamma}}T - 2D_{\tilde{\gamma}} \leq 0$ then $\frac{\partial \mathcal{R}_0}{\partial \tilde{\gamma}} < 0$. If $T_{\tilde{\gamma}}T - 2D_{\tilde{\gamma}} > 0$, then

$$\begin{aligned}
\frac{\partial \mathcal{R}_0}{\partial \tilde{\gamma}} < 0 \quad &\Leftrightarrow \quad \frac{1}{2}\left[T_{\tilde{\gamma}} + \frac{T_{\tilde{\gamma}}T - 2D_{\tilde{\gamma}}}{\sqrt{T^2 - 4D}}\right] \; < \; 0 \\
&\Leftrightarrow \quad \left[4D_{\tilde{\gamma}} - 2T_{\tilde{\gamma}}T\right]^2 \; < \; 4T_{\tilde{\gamma}}^2\left[T^2 - 4D\right] \\
&\Leftrightarrow \quad T_{\tilde{\gamma}}^2 D \; < \; D_{\tilde{\gamma}}T_{\tilde{\gamma}}T - D_{\tilde{\gamma}}^2 \\
&\Leftrightarrow \quad (\mathcal{R}^{hor})^2\mathcal{R}^{ver}(1-p)p(\mathcal{R}^{hor} - \mathcal{R}^{ver}) < 0 \\
&\Leftrightarrow \quad \mathcal{R}^{hor} < \mathcal{R}^{ver}.
\end{aligned}$$

Thus, if $\mathcal{R}^{hor} < \mathcal{R}^{ver}$ then $\frac{\partial \mathcal{R}_0}{\partial \tilde{\gamma}} < 0$. This means the basic reproduction number is a strictly monotone decreasing function of γ when $\mathcal{R}^{hor} < \mathcal{R}^{ver}$. Thus, by the continuity of $\mathcal{R}_0$ is maximized at $\gamma^* = \gamma_{min}$ and minimized at $\gamma = \gamma_{max}$ uniquely. □

C.2 Maximization of $\mathcal{R}_0$ and feasible invasion strategies

Using lemma (C.2) and lemma (C.3), we summarize the results of maximal viral strategy in the following theory.

Theorem 1 *The basic reproduction number $\mathcal{R}_0(p,\gamma)$, Eq. [B.27], is maximized at $p^* = 0$ and all $\gamma_{min} \leq \gamma \leq \gamma_{max}$ if $\mathcal{R}^{hor} > \mathcal{R}^{ver}$. On the other hand, $\mathcal{R}_0(p,\gamma)$ is maximized uniquely at $(p^*,\gamma^*) = (1,\gamma_{min})$ if $\mathcal{R}^{hor} < \mathcal{R}^{ver}$.*

Proof. The proof follows from lemma (C.2) and lemma (C.3). If $\mathcal{R}^{hor} > \mathcal{R}^{ver}$, then

$$\begin{aligned}
\max_{\gamma\in[\gamma_{min},\gamma_{max}]}\ \max_{p\in[0,1]} \mathcal{R}_0(p,\gamma) &= \max_{\gamma\in[\gamma_{min},\gamma_{max}]} \mathcal{R}_0(p^* = 0,\gamma) \\
&= \max_{\gamma\in[\gamma_{min},\gamma_{max}]} \max\{\mathcal{R}^{hor},\ (1-\tilde{\gamma})\mathcal{R}^{ver}\} \\
&= \mathcal{R}^{hor} \qquad \forall\ \gamma \in [\gamma_{min},\gamma_{max}].
\end{aligned}$$

On the other hand, if $\mathcal{R}^{hor} < \mathcal{R}^{ver}$, then

$$\begin{aligned}
\max_{\gamma\in[\gamma_{min},\gamma_{max}]}\ \max_{p\in[0,1]} \mathcal{R}_0(p,\gamma) &= \max_{\gamma\in[\gamma_{min},\gamma_{max}]} \mathcal{R}_0(p^* = 1,\gamma) \\
&= \max_{\gamma\in[\gamma_{min},\gamma_{max}]} \tilde{\gamma}\mathcal{R}^{hor} + (1-\tilde{\gamma})\mathcal{R}^{ver} \\
&= \mathcal{R}_0(1,\gamma_{min}).
\end{aligned}$$

□

Remark 1 *We note the following:*
(i) $\mathcal{R}_0(p,\gamma)$ is bounded above by $max(\mathcal{R}^{hor},\mathcal{R}^{ver})$ and bounded below by $min(\mathcal{R}^{hor},\mathcal{R}^{ver})$.
(ii) If $\mathcal{R}^{hor} = \mathcal{R}^{ver}$, then $\mathcal{R}_0(p,\gamma) = \mathcal{R}^{hor}$ for all $(p,\gamma) \in \Theta$.
(iii) If $min(\mathcal{R}^{hor},\mathcal{R}^{ver}) > 1$, then $\mathcal{R}_0(p,\gamma) > 1$ for all $(p,\gamma) \in \Theta$.
(iv) If $max(\mathcal{R}^{hor},\mathcal{R}^{ver}) < 1$, then $\mathcal{R}_0(p,\gamma) < 1$ for all $(p,\gamma) \in \Theta$.
(v) If $max(\mathcal{R}^{hor},\mathcal{R}_0(1,\gamma_{min})) > 1$ and $min(\mathcal{R}^{hor},\mathcal{R}_0(1,\gamma_{min})) < 1$, then there exists a critical curve $\mathcal{C} = \{(p,\gamma) : \mathcal{R}_0(p,\gamma) = 1\}$, which partitions the viral strategy space Θ into feasible strategy regime (i.e., $\mathcal{R}_0(p,\gamma) > 1$) and infeasible strategy regime (i.e., $\mathcal{R}_0(p,\gamma) < 1$).

Remark 2 *For the two resource-implicit models [A.1] and [A.3], theorem (1) can be restated in terms of the density of susceptible hosts at the virus-free equilibrium (S^*). Specifically, because $\mathcal{R}^{hor}$ is a monotonically increasing function of S^* and $\mathcal{R}^{ver}$ is a monotonically decreasing function of S^*, there exists a critical value S_c that satisfies $\mathcal{R}^{hor}(S_c) = \mathcal{R}^{ver}(S_c)$. Thus, we can restate theorem (1) in terms of S^*: If $S^* > S_c$, then $\mathcal{R}^{hor} > \mathcal{R}^{ver}$ and the maximal strategy is $p^* = 0$ and arbitrary $\gamma \in [\gamma_{min},\gamma_{max}]$. If $S^* < S_c$, then $\mathcal{R}^{hor} < \mathcal{R}^{ver}$ and the maximal strategy is $p^* = 1$ and $\gamma = \gamma_{min}$.*

Remark 3 *For the two resource-explicit models [A.2] and [A.4], theorem (1) can be restated in terms of the resource concentration and the density of susceptible hosts at the virus-free equilibrium* (R^*, S^*). *Specifically, because* $\mathcal{R}^{hor}$ *is a monotonically increasing function of* S^* *and* $\mathcal{R}^{ver}$ *is a monotonically increasing function of* R^*, *there exists a curve in* R^*, S^**-space where there is a transition from the purely lytic strategy* $(p = 0)$ *maximizing* $\mathcal{R}_0$ *to the purely lysogenic strategy* $(p = 1, \gamma = \gamma_{min})$ *maximizing* $\mathcal{R}_0$. *That curve is defined by* $\Gamma = \{(R^*, S^*) : \mathcal{R}^{hor}(S^*) - \mathcal{R}^{ver}(R^*) = 0\}$. *We decompose* R^*, S^**-space into two regions based on* Γ, *let* $\Gamma_+ = \{(R^*, S^*) : \mathcal{R}^{hor}(S^*) - \mathcal{R}^{ver}(R^*) > 0\}$ *and* $\Gamma_- = \{(R^*, S^*) : \mathcal{R}^{hor}(S^*) - \mathcal{R}^{ver}(R^*) < 0\}$. *Thus, we can restate theorem (1) in terms of* R^* *and* S^*: *If* $(R^*, S^*) \in \Gamma_+$, *then* $\mathcal{R}^{hor} > \mathcal{R}^{ver}$ *and the maximal strategy is* $p^* = 0$ *and arbitrary* $\gamma \in [\gamma_{min}, \gamma_{max}]$. *If* $(R^*, S^*) \in \Gamma_-$, *then* $\mathcal{R}^{hor} < \mathcal{R}^{ver}$ *and the maximal strategy is* $p^* = 1$ *and* $\gamma = \gamma_{min}$.

Remark 4 *Let* σ *denote the largest eigenvalue of the linearized infected subsystem,* $\mathcal{J}$. *Biologically,* σ *is the exponential growth rate of viral strategy in a virus-free host population. Both* σ *and* $\mathcal{R}_0$ *can be used to predict viral invasion because* $sign\{\sigma\} = sign\{\mathcal{R}_0 - 1\}$. *The values of* σ *and* $\mathcal{R}_0$ *may not be maximized by the same viral strategy. We numerically explored what viral strategies maximized* σ *in model [A.1]. Our numerical results showed that there exists a critical density of susceptible hosts,* S_c', *such that if* $S^* > S_c'$ *then* σ *is maximized at* $p^* = 0$ *and arbitrary* $\gamma \in (\gamma_{min}, \gamma_{max})$ *and if* $S^* < S_c'$ *then* σ *is maximized at* $p^* = 1$ *and* $\gamma = \gamma_{min}$. *However, the critical values* S_c *and* S_c' *are not the same; see Table (1) for an example. Thus, in general, the viral strategy that maximizes* $\mathcal{R}_0$ *is not the strategy that maximizes instantaneous growth rate.*

D Endemically Infected States and Viral Invasion

In this section, we provide details about the evolutionary analysis for the four model variants. In doing so, we apply an invasion analyses (Hurford et al. (2010); Wahl et al. (2019)), in which the system reaches a stable endemic equilibrium with a resident viral strategy $(p_r, \gamma_r) \in \Theta$. We then introduce a mutant viral strain with strategy $(p_m, \gamma_m) \in \Theta$ and determine if the mutant can invade the resident equilibrium. We start by deriving the next-generation matrix of the mutant at the resident equilibrium.

D.1 Mutual invasion analysis via NGM approach

The mutant-resident system of model [A.1] is

$$\begin{aligned}
\dot{S} &= r_S S\left(1 - \frac{N}{K}\right) - \phi S V - \phi S V_m - d_S S \\
\dot{E} &= \phi S V - (\lambda + d_E) E \\
\dot{L} &= p_r \lambda E + r_L L\left(1 - \frac{N}{K}\right) - (\gamma_r + d_L) L \\
\dot{I} &= (1 - p_r)\lambda E + \gamma_r L - (\eta + d_I) I \\
\dot{V} &= \beta \eta I - \phi N V - m V \\
\dot{E}_m &= \phi S V_m - (\lambda + d_E) E_m \\
\dot{L}_m &= p_m \lambda E_m + r_L L_m\left(1 - \frac{N}{K}\right) - (\gamma_m + d_L) L_m \\
\dot{I}_m &= (1 - p_m)\lambda E_m + \gamma_m L_m - (\eta + d_I) I_m \\
\dot{V}_m &= \beta \eta I_m - \phi N V_m - m V_m
\end{aligned} \tag{D.29}$$

where $N = S + E + L + I + E_m + L_m + I_m$ is the total population of hosts. The E_m, L_m, I_m, V_m-subsystem denotes the classes with the mutant viral strategies. The resident endemic equilibrium of system [D.29] is $(S^+, E^+, L^+, I^+, V^+, 0, 0, 0, 0)$. Invasion of the resident endemic equilibrium is determined by the magnitude of the maximum eigenvalue of the next generation matrix,

$$\Phi_r^m = \begin{bmatrix} \mathcal{R}_+^{hor}\widetilde{\gamma_m} p_m + (1 - p_m)\mathcal{R}_+^{hor} & \mathcal{R}_+^{hor}(\widetilde{\gamma}_m/\widetilde{\lambda}) \\ \mathcal{R}_+^{ver} p_m \widetilde{\lambda}(1 - \widetilde{\gamma}_m) & \mathcal{R}_+^{ver}(1 - \widetilde{\gamma}_m) \end{bmatrix} \tag{D.30}$$

where

$$\widetilde{\gamma_m} = \frac{\gamma_m}{\gamma_m + d_L}, \ \widetilde{\lambda} = \frac{\lambda}{\lambda + d_E}, \ \mathcal{R}_+^{hor}(S^+, N^+) = \frac{\beta\eta\phi S^+\lambda}{(\eta + d_I)(\phi N^+ + m)(\lambda + d_E)}, \ \mathcal{R}_+^{ver}(N^+) = \frac{r_L\left(1 - \frac{N^+}{K}\right)}{d_L} \tag{D.31}$$

and $N^+ = S^+ + E^+ + L^+ + I^+$. Note that $\mathcal{R}_+^{hor}$ has two inputs, we define $\mathcal{R}_+^{hor}(x,y) = \frac{\beta\eta\phi x\lambda}{(\eta+d_I)(\phi y+m)(\lambda+d_E)}$.

The largest eigenvalue of Φ_r^m (mutant invasion fitness) is denoted by $\mathcal{R}_{inv}(p_m, \gamma_m|p_r, \gamma_r)$. The sign of $\mathcal{R}_{inv}(p_m, \gamma_m|p_r, \gamma_r) - 1$ determines the invasion of the resident endemic equilibrium in system [D.29].

Similarly, we add the L_m, V_m-subsystem to model [A.3]. The resident endemic equilibrium of the augmented resident-mutant system of model [A.3] is denoted by $(S^+, L^+, V^+, 0, 0)$. The next-generation matrix of a mutant strain with viral strategy (p_m, γ_m) is

$$\Phi_r^m = \begin{bmatrix} \mathcal{R}_+^{hor}\widetilde{\gamma_m}p_m + (1-\widetilde{\gamma_m})\mathcal{R}_+^{ver} & \mathcal{R}_+^{hor}(p_m/\beta) \\ \mathcal{R}_+^{hor}(1-p_m)\widetilde{\gamma_m}\beta & \mathcal{R}_+^{hor}(1-p_m) \end{bmatrix} \tag{D.32}$$

where

$$\widetilde{\gamma_m} = \frac{\gamma_m}{\gamma_m + m}, \quad \mathcal{R}_+^{hor} = \frac{b\beta\phi S^+}{\phi N^+ + m}, \quad \mathcal{R}_+^{ver} = \frac{r_L\delta}{m}\left(1 - \frac{N^+}{K}\right) \tag{D.33}$$

and $N^+ = S^+ + L^+$.

Again, we add the E_m, L_m, I_m, V_m-subsystem to model [A.2]. The resident endemic equilibrium of the augmented resident-mutant system of model [A.2] is denoted by $(R^+, S^+, E^+, L^+, I^+, V^+, 0, 0, 0, 0)$. The next-generation matrix of a mutant strain with viral strategy (p_m, γ_m) is

$$\Phi_r^m = \begin{bmatrix} \mathcal{R}_+^{hor}\widetilde{\gamma_m}p_m + (1-p_m)\mathcal{R}_+^{hor} & \mathcal{R}_+^{hor}(\widetilde{\gamma_m}/\widetilde{\lambda}) \\ \mathcal{R}_+^{ver}p_m\widetilde{\lambda}(1-\widetilde{\gamma_m}) & \mathcal{R}_+^{ver}(1-\widetilde{\gamma_m}) \end{bmatrix} \tag{D.34}$$

where

$$\widetilde{\gamma_m} = \frac{\gamma_m}{\gamma_m + d_L}, \ \widetilde{\lambda} = \frac{\lambda}{\lambda + d_E} \ \mathcal{R}_+^{hor}(S^+, N^+) = \frac{\beta\eta\phi S^+\lambda}{(\eta + d_I)(\phi N^+ + m)(\lambda + d_E)}, \ \mathcal{R}_+^{ver}(R^+) = \frac{\psi(R^+)}{d_L}. \tag{D.35}$$

Finally, we add the L_m, V_m-subsystem to model [A.4]. The resident endemic equilibrium of the augmented resident-mutant system of model [A.4] is denoted by $(R^+, S^+, L^+, V^+, 0, 0)$. The next-generation matrix of a mutant strain with viral strategy (p_m, γ_m) is

$$\Phi_r^m = \begin{bmatrix} \mathcal{R}_+^{hor}\widetilde{\gamma_m}p_m + (1-\widetilde{\gamma_m})\mathcal{R}_+^{ver} & \mathcal{R}_+^{hor}(p_m/\beta) \\ \mathcal{R}_+^{hor}(1-p_m)\widetilde{\gamma_m}\beta & \mathcal{R}_+^{hor}(1-p_m) \end{bmatrix} \tag{D.36}$$

where

$$\widetilde{\gamma_m} = \frac{\gamma_m}{\gamma_m + \rho + \nu}, \quad \mathcal{R}_+^{hor}(S^+) = \frac{\phi S^+\beta}{\rho + \phi L^+}, \quad \mathcal{R}_+^{ver}(R^+) = \frac{\psi(R^+)}{\rho + \nu}. \tag{D.37}$$

Notably, the derived next-generation matrices for the mutant strains in all four model variants are 2×2 matrices. The traces, $T(\Phi_r^m)$, and determinants, $D(\Phi_r^m)$ of four next-generation matrices are written as

$$T(\Phi_r^m) = \mathcal{R}_+^{hor}\widetilde{\gamma_m}p_m + (1-p_m)\mathcal{R}_+^{hor} + (1-\widetilde{\gamma_m})\mathcal{R}_+^{ver}, \quad D(\Phi_r^m) = \mathcal{R}_+^{hor}\mathcal{R}_+^{ver}(1-p_m)(1-\widetilde{\gamma_m}), \tag{D.38}$$

where the $\mathcal{R}_+^{hor}, \mathcal{R}_+^{ver}$ and $\widetilde{\gamma_m}$ are given in Eq. [D.31], Eq. [D.33], Eq. [D.35] and Eq. [D.37]. The dominate eigenvalue $\mathcal{R}_{inv}(p_m, \gamma_m|p_r, \gamma_r)$ can be obtained from the trace and the determinant of the next-generation matrix,

$$\mathcal{R}_{inv}(p_m, \gamma_m|p_r, \gamma_r) = \frac{1}{2}\left(T(\Phi_r^m) + \sqrt{T(\Phi_r^m)^2 - 4D(\Phi_r^m)}\right). \tag{D.39}$$

Using the fact that $\mathcal{R}_{inv}(p_m, \gamma_m|p_r, \gamma_r) = 1$ when $(p_m, \gamma_m) = (p_r, \gamma_r)$, and the monotonicity of $\mathcal{R}_{inv}(p_m, \gamma_m|p_r, \gamma_r)$ on $(p_m, \gamma_m) \in \Theta$ provided by lemma (C.2), lemma (C.3) and theorem (1), we immediately obtain the following theorem.

Theorem 2 *For all of the four model variants, we assume the susceptible cell population is positive at the stable resident endemic equilibrium, i.e.,* $S^+ > 0$*. Then, we have:*

(i) If $\mathcal{R}_+^{hor} < \mathcal{R}_+^{ver}$*, viral strains with more lysogenic strategies (i.e.,* $p_r < p_m \le 1$ *and* $\gamma_{min} \le \gamma_m < \gamma_r$*) can invade, and viral strains with more lytic strategies (i.e.,* $0 \le p_m < p_r$ *and* $\gamma_r < \gamma_m \le \gamma_{max}$*) cannot invade.*

(ii) If $\mathcal{R}_+^{hor} > \mathcal{R}_+^{ver}$*, viral strains with more lysogenic strategies (i.e.,* $p_r < p_m \le 1$ *and* $\gamma_{min} \le \gamma_m < \gamma_r$*) cannot invade, and viral strains with more lytic strategies (i.e.,* $0 \le p_m < p_r$ *and* $\gamma_r < \gamma_m \le \gamma_{max}$*) can invade.*

Remark 5 *For model [A.1] and model [A.2], the stable resident endemic equilibrium might be a boundary equilibrium with* $S^+ = 0$*. If the resident system reaches a stable endemic equilibrium with* $S^+ = 0$*, then no mutant strain can invade. Specifically, a mutant strain is introduced at the resident equilibrium through a virus particle, the first step in the proliferation of a virus is that it infects a susceptible cell, hence, a virus mutant cannot spread given the absence of a susceptible population.*

D.2 Robustness of maximal strategies

Theorem (2) requires the specification of the resident equilibrium to determine whether mutant invasion scenarios correspond to case (i) or case (ii). In this section, we provide additional results to apply theorem (2) to model [A.1] and model [A.2]. Further, we assume the resident strain is either the purely lytic ($p = 0$) or purely lysogenic strategy ($p = 1, \gamma = \gamma_{min}$).

Proposition D.1 *(i) The stable resident endemic equilibrium of model [A.1],* $(S^+, E^+, L^+, I^+, V^+)$ *with* $S^+ > 0$*, satisfies* $S^+ < N^+ < S^*$*, where* S^* *is given by Eq. [B.10].*

(ii) The stable resident endemic equilibrium of model [A.2], $(R^+, S^+, E^+, L^+, I^+, V^+)$ *with* $S^+ > 0$*, satisfies* $R^+ > R^*$ *and* $S^+ < S^*$*, where* R^* *and* S^* *are given by Eq. [B.12].*

Proof. Proof of (i) : $S^+ < N^+$ is a trivial observation. We prove $N^+ < S^*$ by contradiction. Suppose $N^+ \ge S^*$ and set $\dot{S} = 0$ in model [A.1]

$$0 = S^+ \left[r_S \left(1 - \frac{N^+}{K} \right) - d_S \right] - \phi S^+ V^+ \le -\phi S^+ V^+ < 0 \ .$$

This shows a contradiction such that (i) is true.

Proof of (ii) : At the stable resident endemic equilibrium of model [A.2], we set $\dot{R} = 0$ and $\dot{S} = 0$

$$\begin{cases} 0 = & J - d_R R^+ - e\psi(R^+)\left(L^+ + (1-\alpha_s)S^+\right) \\ 0 = & S^+\left[(1-\alpha_s)\psi(R^+) - \phi V^+ - d_S\right] \end{cases}$$

Given $S^+ > 0$, we rewrite the above system and find that

$$S^+ = \frac{(J - d_R R^+) - e\psi(R^+)L^+}{e(d_S + \phi V^+)}, \quad \psi(R^+) = \frac{\phi V^+ + d_S}{1 - \alpha_s}$$

Note that $\psi(R^*) = d_S/(1-\alpha_s)$ by substituting virus-free equilibrium, Eq. [B.12], into the Monod equation. Then, we find that

$$\psi(R^+) = \frac{\phi V^+ + d_S}{1 - \alpha_s} > \frac{d_S}{1 - \alpha_s} = \psi(R^*).$$

It should be clear that $R^+ > R^*$ since ψ, the Monod equation, is a strictly monotone increasing function. In addition,

$$S^+ = \frac{(J - d_R R^+) - e\psi(R^+)L^+}{e(d_S + \phi V^+)} < \frac{(J - d_R R^*)}{e d_S} = S^*.$$

□

Proposition D.2 *(i) If the virus-free equilibrium of model [A.1] satisfies* $\mathcal{R}^{hor}(S^*) < \mathcal{R}^{ver}(S^*)$*, where* $\mathcal{R}^{hor}(S^*)$ *and* $\mathcal{R}^{ver}(S^*)$ *are given by Eq. [B.16], then* $\mathcal{R}_+^{hor}(S^+, N^+) < \mathcal{R}_+^{ver}(N^+)$ *at the stable resident endemic equilibrium, where* $\mathcal{R}_+^{hor}(S^+, N^+)$ *and* $\mathcal{R}_+^{ver}(N^+)$ *are given by Eq. [D.31].*

(ii) If the virus-free equilibrium of model [A.2] satisfies $\mathcal{R}^{hor}(S^*) < \mathcal{R}^{ver}(R^*)$*, where* $\mathcal{R}^{hor}$ *and* $\mathcal{R}^{ver}$ *are given by Eq. [B.22], then* $\mathcal{R}_+^{hor}(S^+, N^+) < \mathcal{R}_+^{ver}(R^+)$ *at the stable resident endemic equilibrium, where* $\mathcal{R}_+^{hor}(S^+, N^+)$ *and* $\mathcal{R}_+^{ver}(R^+)$ *are given by Eq. [D.35].*

Proof. Proof of (*i*): First, we assume that the susceptible cells population at resident equilibrium of model [A.1] is positive, $S^+ > 0$, then we have $S^+ < N^+ < S^*$ by proposition (D.1). Given $\mathcal{R}^{hor}(S^*) < \mathcal{R}^{ver}(S^*)$ in Eq. [B.16], we arrive

$$\mathcal{R}_+^{ver}(N^+) > \mathcal{R}^{ver}(S^*) > \mathcal{R}^{hor}(S^*) > \mathcal{R}_+^{hor}(S^+, S^+) > \mathcal{R}_+^{hor}(S^+, N^+).$$

Second, if $S^+ = 0$ at the resident equilibrium of model [A.1], $\mathcal{R}_+^{hor} = 0$, it's clear that $\mathcal{R}_+^{ver} > \mathcal{R}_+^{hor}$.

Proof of (*ii*): First, we assume that the susceptible cells population at resident equilibrium of model [A.2] is positive, $S^+ > 0$, then we have $S^+ < S^*$ and $R^+ > R^*$ by proposition (D.1). Given $\mathcal{R}^{hor}(S^*) < \mathcal{R}^{ver}(R^*)$ in Eq. [B.22], we arrive

$$\mathcal{R}_+^{ver}(R^+) > \mathcal{R}^{ver}(R^*) > \mathcal{R}^{hor}(S^*) > \mathcal{R}_+^{hor}(S^+, S^+) > \mathcal{R}_+^{hor}(S^+, N^+).$$

Second, if $S^+ = 0$ at the resident equilibrium of model [A.2], $\mathcal{R}_+^{hor} = 0$, it's clear that $\mathcal{R}_+^{ver} > \mathcal{R}_+^{hor}$. □

For model [A.1] and model [A.2], when the resident strains are either the purely lytic strategy ($p = 0$) or the purely lysogenic strategy ($p = 1, \gamma = \gamma_{min}$), some qualitative results of evolutionary invasion are immediately obtained by theorem (2) and proposition (D.2). First, in the event that resident strains are purely lytic ($p = 0$), then, we have remark (6).

Remark 6 *We assume the resident viral strain is the purely lytic strategy ($p = 0$).*

(i) If the virus-free equilibrium of model [A.1] satisfies $\mathcal{R}^{hor}(S^*) < \mathcal{R}^{ver}(S^*)$, where $\mathcal{R}^{hor}(S^*)$ and $\mathcal{R}^{ver}(S^*)$ are given by Eq. [B.16], the viral strains with more lysogenic strategies (i.e., $0 < p_m \leq 1$) can invade the stable resident endemic equilibrium.

(ii) If the virus-free equilibrium of model [A.1] satisfies $\mathcal{R}^{hor}(S^*) > \mathcal{R}^{ver}(S^*)$, where $\mathcal{R}^{hor}(S^*)$ and $\mathcal{R}^{ver}(S^*)$ are given by Eq. [B.16], the viral strains with more lysogenic strategies (i.e., $0 < p_m \leq 1$) can invade the stable resident endemic equilibrium if $\mathcal{R}_+^{hor} < \mathcal{R}_+^{ver}$, where $\mathcal{R}_+^{hor}$ and $\mathcal{R}_+^{ver}$ are given by Eq. [D.31]. In contrast, the viral strains with more lysogenic strategies cannot invade the stable resident endemic equilibrium if $\mathcal{R}_+^{hor} > \mathcal{R}_+^{ver}$.

(iii) If the virus-free equilibrium of model [A.2] satisfies $\mathcal{R}^{hor}(S^*) < \mathcal{R}^{ver}(R^*)$, where $\mathcal{R}^{hor}(S^*)$ and $\mathcal{R}^{ver}(R^*)$ are given by Eq. [B.22], the viral strains with more lysogenic strategies (i.e., $0 < p_m \leq 1$) can invade the stable resident endemic equilibrium.

(iv) If the virus-free equilibrium of model [A.2] satisfies $\mathcal{R}^{hor}(S^*) > \mathcal{R}^{ver}(R^*)$, where $\mathcal{R}^{hor}(S^*)$ and $\mathcal{R}^{ver}(R^*)$ are given by Eq. [B.22], the viral strains with more lysogenic strategies (i.e., $0 < p_m \leq 1$) can invade the stable resident endemic equilibrium if $\mathcal{R}_+^{hor} < \mathcal{R}_+^{ver}$, where $\mathcal{R}_+^{hor}$ and $\mathcal{R}_+^{ver}$ are given by Eq. [D.35]. In contrast, the viral strains with more lysogenic strategies cannot invade the stable resident endemic equilibrium if $\mathcal{R}_+^{hor} > \mathcal{R}_+^{ver}$.

Second, in the event that the resident strain is purely lysogenic ($p = 1, \gamma = \gamma_{min}$) and the susceptible cell population is positive at the resident equilibrium, $S^+ > 0$, then we arrive at remark (7). Note that the invasion scenario of $S^+ = 0$ is provided in remark (5).

Remark 7 *We assume the resident viral strain is the purely lysogenic strategy ($p = 1, \gamma = \gamma_{min}$) and the susceptible cell population is positive at the resident equilibrium, $S^+ > 0$.*

(i) If the virus-free equilibrium of model [A.1] satisfies $\mathcal{R}^{hor}(S^*) < \mathcal{R}^{ver}(S^*)$, where $\mathcal{R}^{hor}(S^*)$ and $\mathcal{R}^{ver}(S^*)$ are given by Eq. [B.16], the viral strains with more lytic strategies (i.e., $0 \leq p_m < 1$ and $\gamma_{min} < \gamma_m \leq \gamma_{max}$) cannot invade the stable resident endemic equilibrium.

(ii) If the virus-free equilibrium of model [A.1] satisfies $\mathcal{R}^{hor}(S^*) > \mathcal{R}^{ver}(S^*)$, where $\mathcal{R}^{hor}(S^*)$ and $\mathcal{R}^{ver}(S^*)$ are given by Eq. [B.16], the viral strains with more lytic strategies (i.e., $0 \leq p_m < 1$ and $\gamma_{min} < \gamma_m \leq \gamma_{max}$) can invade the stable resident endemic equilibrium if $\mathcal{R}_+^{hor} > \mathcal{R}_+^{ver}$, where $\mathcal{R}_+^{hor}$ and $\mathcal{R}_+^{ver}$ are given by Eq. [D.31]. In contrast, the viral strains with more lytic strategies cannot invade the stable resident endemic equilibrium if $\mathcal{R}_+^{hor} < \mathcal{R}_+^{ver}$.

(iii) If the virus-free equilibrium of model [A.2] satisfies $\mathcal{R}^{hor}(S^*) < \mathcal{R}^{ver}(R^*)$, where $\mathcal{R}^{hor}(S^*)$ and $\mathcal{R}^{ver}(R^*)$ are given by Eq. [B.22], the viral strains with more lytic strategies (i.e., $0 \leq p_m < 1$ and $\gamma_{min} < \gamma_m \leq \gamma_{max}$) cannot invade the stable resident endemic equilibrium.

(iv) If the virus-free equilibrium of model [A.2] satisfies $\mathcal{R}^{hor}(S^*) > \mathcal{R}^{ver}(R^*)$, where $\mathcal{R}^{hor}(S^*)$ and $\mathcal{R}^{ver}(R^*)$ are given by Eq. [B.22], the viral strains with more lytic strategies (i.e., $0 \leq p_m < 1$ and $\gamma_{min} < \gamma_m \leq \gamma_{max}$) can invade the stable resident endemic equilibrium if $\mathcal{R}_+^{hor} > \mathcal{R}_+^{ver}$, where $\mathcal{R}_+^{hor}$ and $\mathcal{R}_+^{ver}$ are given by Eq. [D.35]. In contrast, the viral strains with more lytic strategies cannot invade the stable resident endemic equilibrium if $\mathcal{R}_+^{hor} < \mathcal{R}_+^{ver}$.

By theorem (1), the maximal strategies, i.e., the strategies that maximize $\mathcal{R}_0$, are either purely lytic strategy or the purely lysogenic strategy. For model [A.1] and model [A.2], when the resident strains are the maximal strategies, we provide remark (8).

Remark 8 *(i) If the purely lysogenic strategy maximizes $\mathcal{R}_0$, then no other strategy can invade the endemic equilibrium for the purely lysogenic strategy.*

(ii) If the purely lytic strategy maximizes $\mathcal{R}_0$, then no other strategy can invade the endemic equilibrium for the purely lytic strategy, provided $\mathcal{R}_+^{hor} > \mathcal{R}_+^{ver}$ holds. Otherwise, if $\mathcal{R}_+^{hor} < \mathcal{R}_+^{ver}$, the endemic equilibrium for the purely lytic strategy can be invaded by viral strains with more temperate strategies (i.e., $p_r < p_m \leq 1$ and $\gamma_{min} \leq \gamma_m < \gamma_r$).

D.3 Invasion of temperate phage with varying degrees of super-infection immunity

In this section, we detail the invasion analysis for model [A.2] that includes super-infection. In order to control the degree of super-infection immunity, we introduce a new parameter ϵ, where $0 \leq \epsilon \leq 1$. When $\epsilon = 1$, lysogens have full super immunity, viruses are absorbed into all the cells but only actively infect susceptible cells (i.e., super-infection is a 'sink' for viruses). When $\epsilon = 0$, purely lytic viruses are absorbed into all cells and can switch a lysogen into an actively infected cell. We consider the following mutant-resident system of model [A.2] with the super-infection immunity mechanism (assuming resident strain is purely lytic ($p = 0$), while the mutant strain is temperate with $p_m > 0$)

$$\begin{aligned}
\dot{R} &= J - d_R R - f(R, S, L_m) \\
\dot{S} &= (1 - \alpha_S)\psi(R)S - \phi SV - \phi SV_m - d_S S \\
\dot{E} &= \phi SV + (1-\epsilon)\phi L_m V - (\lambda + d_E)E \\
\dot{I} &= \lambda E - (\eta + d_I)I \\
\dot{V} &= \beta\eta I - \phi NV - mV \\
\dot{E}_m &= \phi SV_m - (\lambda + d_E)E_m \\
\dot{L}_m &= p_m\lambda E_m + \psi(L_m)L_m - (1-\epsilon)\phi L_m V - (\gamma_m + d_L)L_m \\
\dot{I}_m &= (1 - p_m)\lambda E_m + \gamma_m L_m - (\eta + d_I)I_m \\
\dot{V}_m &= \beta\eta I_m - \phi NV_m - mV_m
\end{aligned} \tag{D.40}$$

where $f(R, S, L_m) = e\psi(R)\left(L_m + (1 - \alpha_S)S\right)$ denotes the cumulative uptake of nutrients by all cells and $N = S + E + I + E_m + L_m + I_m$ is the total population of hosts. The next-generation matrix of a mutant strain with viral strategy (p_m, γ_m) is

$$\Phi_r^m = \begin{bmatrix} \mathcal{R}_+^{hor}\widetilde{\gamma_m}p_m + (1-p_m)\mathcal{R}_+^{hor} & \mathcal{R}_+^{hor}(\widetilde{\gamma_m}/\widetilde{\lambda}) \\ \mathcal{R}_+^{ver}p_m\widetilde{\lambda}(1-\widetilde{\gamma_m}) & \mathcal{R}_+^{ver}(1-\widetilde{\gamma_m}) \end{bmatrix} \tag{D.41}$$

where $\widetilde{\gamma_m} = \frac{\gamma_m}{\gamma_m + d_L}$, $\widetilde{\lambda} = \frac{\lambda}{\lambda + d_E}$ and

$$\mathcal{R}_+^{hor}(S^+, N^+) = \frac{\beta\eta\phi S^+\lambda}{(\eta + d_I)(\phi N^+ + m)(\lambda + d_E)} \text{ , } \mathcal{R}_+^{ver}(R^+) = \frac{\psi(R^+)}{d_L + (1-\epsilon)\phi V^+}. \tag{D.42}$$

The mutant invasion fitness $\mathcal{R}_{inv}$ is the largest eigenvalue of NGM Φ_r^m presented in Eq. [D.41].

E Model Parameters

Here we present the parameters used to support the figures presented in this study.

Table 1: Parameters of model (A.1), source from (Weitz (2015), Weitz et al. (2019))

Parameters	Value	Unit
r_S, maximal cellular growth rate of susceptible cells	$0.2 + 10^{-4} \sim 1$	h^{-1}
r_L, maximal cellular growth rate of lysogens (with direct benefits)	1.1	h^{-1}
r_L, maximal cellular growth rate of lysogens (with indirect benefits)	0.1	h^{-1}
r_S, maximal cellular growth rate of susceptible cells	$0.2 + 10^{-4} \sim 1$	h^{-1}
d_E, cellular death rate of exposed infected cells	0.2	h^{-1}
d_L, cellular death rate of lysogens	0.2	h^{-1}
d_I, cellular death rate of lytic infected cells	0.2	h^{-1}
K, carrying capacity	2×10^8	ml^{-1}
λ, transition rate from exposed state to fated determined states	2	h^{-1}
η, lysis rate	1	h^{-1}
β, burst size	50	
ϕ, adsorption rate	3.4×10^{-10}	ml/h
m, virion decay rate	1/24	h^{-1}

Table 2: Parameters of model (A.2), source from (Stewart and Levin (1984), Weitz et al. (2019))

Parameters	Value	Unit
e, conversion efficiency	5×10^{-7}	μg
d_R, decay rate of resources	0.5	h^{-1}
μ_{max}, maximum growth rate of cells	1.2	h^{-1}
R_{in}, half-saturation constant	5.0	μg/ml
d_E, cellular death rate of exposed infected cells	0.2	h^{-1}
d_L, cellular death rate of lysogens (with direct benefits)	0.08	h^{-1}
d_L, cellular death rate of lysogens (with indirect benefits)	0.5	h^{-1}
d_I, cellular death rate of lytic infected cells	0.2	h^{-1}
λ, transition rate from exposed state to fated determined states	2	h^{-1}
η, lysis rate	1	h^{-1}
β, burst size	50	
ϕ, adsorption rate	3.4×10^{-10}	ml/h
m, virion decay rate	1/24	h^{-1}
α_S, selection coefficient	$-1 \sim 0.5$	
J, influx rate of resources	$0.23 \sim 11.25$	(μg/ ml) h^{-1}

Table 3: Parameters of model (A.3), source from (Berngruber et al. (2013))

Parameters	Value	Unit
r_L, growth rate of infected cells (with direct benefits)	3.0	h^{-1}
r_L, growth rate of infected cells (with indirect benefits)	0.2	h^{-1}
r_S, growth rate of susceptible cells	$0.75 + 10^{-4} \sim 2.25$	h^{-1}
K, carrying capacity of bacteria	10^9	cells/ml
δ, fidelity of vertical transmission	1	
β, burst size	200	phage/cell
m, dilution rate	0.75	h^{-1}
b, probability of fusion after adsorption	10^{-2}	
ϕ, adsorption constant	10^{-8}	$h^{-1}cell^{-1}$

Table 4: Parameters of model (A.4), source from (Stewart and Levin (1984))

Parameters	Value	Unit
e, conversion efficiency	5×10^{-7}	μg/ cell
ρ, flow rate	0.2	h^{-1}
b, maximum growth rate of cells	0.7	h^{-1}
R_{in}, half-saturation constant	4.0	μg/ml
ν, prophage loss rate (with direct benefits)	10^{-3}	(1/cell) h^{-1}
ν, prophage loss rate (with indirect benefits)	1	(1/cell) h^{-1}
β, burst size	100	phage/cell
ϕ, phage adsorption rate	10^{-9}	(ml/phage) h^{-1}
α_S, selection coefficient	$0.1 \sim 0.61$	
C, resource concentration in the reservoir	$0.21 \sim 56.68$	μg/ ml

References

Berngruber, T. W., Froissart, R., Choisy, M., and Gandon, S. Evolution of virulence in emerging epidemics. *PLOS Pathogens*, 9(3):1–8, 03 2013. doi: 10.1371/journal.ppat.1003209.

Diekmann, O., Heesterbeek, J. A. P., and Roberts, M. G. The construction of next-generation matrices for compartmental epidemic models. *Journal of The Royal Society Interface*, 7(47):873–885, 2010. doi: 10.1098/rsif.2009.0386.

Hurford, A., Cownden, D., and Day, T. Next-generation tools for evolutionary invasion analyses. *Journal of The Royal Society Interface*, 7(45):561–571, 2010. doi: 10.1098/rsif.2009.0448.

Levins, R. Discussion paper: the qualitative analysis of partially specified systems. *Annals of the New York Academy of Sciences*, 231(1):123–138, 1974.

Stewart, F. M. and Levin, B. R. The population biology of bacterial viruses: Why be temperate. *Theoretical Population Biology*, 26(1):93 – 117, 1984. ISSN 0040-5809. doi: https://doi.org/10.1016/0040-5809(84)90026-1.

Wahl, L. M., Betti, M. I., Dick, D. W., Pattenden, T., and Puccini, A. J. Evolutionary stability of the lysis-lysogeny decision: Why be virulent? *Evolution*, 73(1):92–98, 2019. doi: 10.1111/evo.13648.

Weitz, J. S. *Quantitative viral ecology: dynamics of viruses and their microbial hosts*, volume 73. Princeton University Press, 2015.

Weitz, J. S., Li, G., Gulbudak, H., Cortez, M. H., and Whitaker, R. J. Viral invasion fitness across a continuum from lysis to latency. *Virus Evolution*, 5(1):vez006, 2019.
